# Enantioselective phase-transfer catalyzed alkylation of 1-methyl-7-methoxy-2-tetralone: an effective route to dezocine

**DOI:** 10.3762/bjoc.14.119

**Published:** 2018-06-11

**Authors:** Ruipeng Li, Zhenren Liu, Liang Chen, Jing Pan, Weicheng Zhou

**Affiliations:** 1 State Key Lab of New Drug & Pharmaceutical Process, Shanghai Key Lab of Anti-Infectives, Shanghai Institute of Pharmaceutical Industry, China State Institute of Pharmaceutical Industry, No. 285, Gebaini Rd., Shanghai 201203, P. R. of China

**Keywords:** alkylation, asymmetric catalysis, cinchonidine, dezocine

## Abstract

In order to prepare asymmetrically (*R*)-(+)-1-(5-bromopentyl)-1-methyl-7-methoxy-2-tetralone (**3a**), a key intermediate of dezocine, 17 cinchona alkaloid-derived catalysts were prepared and screened for the enantioselective alkylation of 1-methyl-7-methoxy-2-tetralone with 1,5-dibromopentane, and the best catalyst (**C7**) was identified. In addition, optimizations of the alkylation were carried out so that the process became practical and effective.

## Introduction

The preparation of enantiomerically pure compounds has become a stringent requirement for pharmaceutical synthesis [1]. In this context, asymmetric catalysis is probably one of the most attractive procedures for the synthesis of active pharmaceutical ingredients (APIs) due to environmental, operational, and economic benefits.

**Scheme 1:**
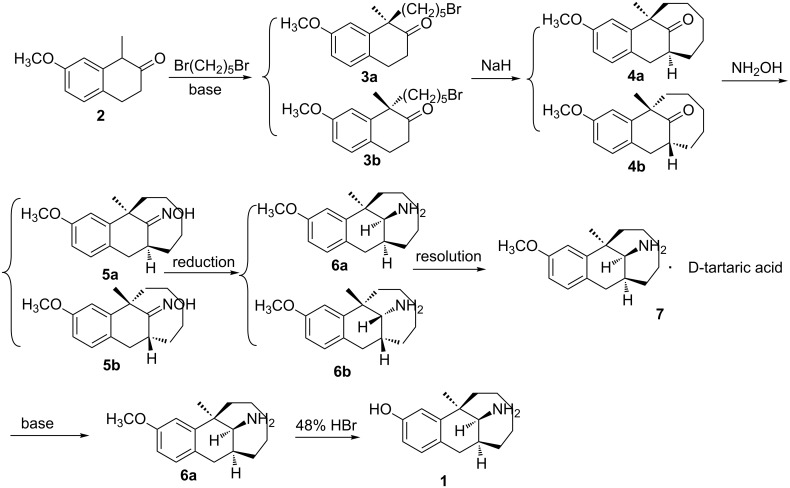
Synthesis of dezocine by resolution.

Dezocine, (5*R*,11*S*,13*S*)-13-amino-5-methyl-5,6,7,8,9,10,11,12-octahydro-5-methyl-5,11-methanobenzocyclodecen-3-ol (**1**, Scheme 1), a typical opioid analgesic developed by AstraZeneca, was extensively used in China recently. Because of its effectiveness and safety [2–3], it would have a very good marketing prospect. However, the cost of dezocine was very high since the commercial synthesis process involved the traditional resolution [4–5]: alkylation of 1-methyl-7-methoxy-2-tetralone (**2**) with 1,5-dibromopentane gave the designed (*R*)-(+)-1-(5-bromopentyl)-1-methyl-7-methoxy-2-tetralone (**3a**) and an equal amount of the *S*-isomer **3b**, both **3a** and **3b** underwent the following cyclization, oximation and reduction, and then, (5*R*,11*S*,13*S*)-3-methoxy-5-methyl-5,6,7,8,9,10,11,12-octahydro-5,11-methanobenzocyclodecen-13-amine (**6a**) and (5*S*,11*R*,13*R*)-3-methoxy-5-methyl-5,6,7,8,9,10,11,12-octahydro-5,11-methanobenzocyclodecen-13-amine (**6b**) were separated by two times of resolution with L-tartaric acid and D-tartaric acid (Scheme 1).

Phase-transfer asymmetric catalysis with cinchona alkaloid-derived quaternary ammonium compounds has become one of the topics in stereoselective synthesis in both industry and academia [6–9]. It was reported [10] that the alkylation of **2** in the catalysis of *N*-(*p*-trifluoromethylbenzyl)cinchonidinium bromide in a two-phase system gave the enantioselective product **3a**, although the ee value of the product was 60%, determined by ^1^H NMR. And so far, no further report on the stereoselective alkylation of **2a** was found. (Some reports on the non-stereoselective alkylation of **2** were given in references [11–12]). In this paper, several cinchona-derived phase-transfer catalysts were screened for this reaction, and the structure–activity relationship for the catalysis was studied. In addition, optimizations had been made to make the process efficient.

## Results and Discussion

**Scheme 2:**
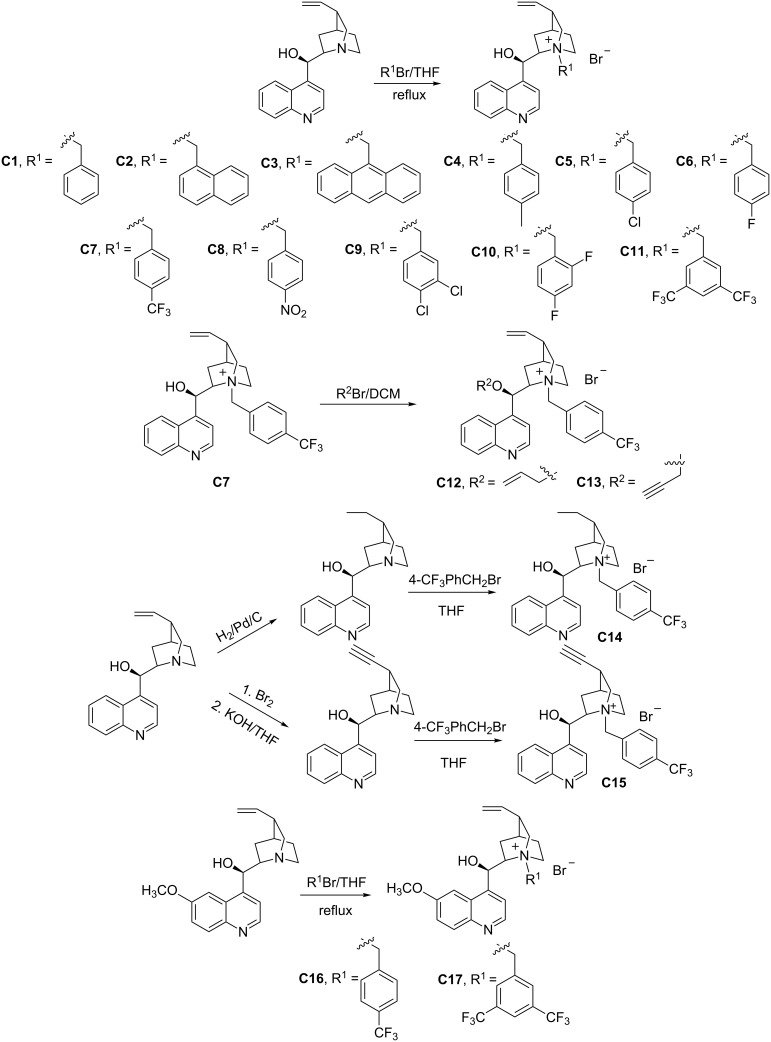
Synthesis of catalysts **C1**–**C17**.

A series of the quaternary ammonium bromides from cinchonidine or quinine as phase-transfer catalysts was prepared (Scheme 2). Cinchonidine was reacted with the benzyl bromides (R^1^Br) in THF to obtain catalysts **C1–C11** [13]. And then **C7** reacted with allyl or propargyl bromide to obtain **C12** and **C13**. In another way, cinchonidine was reduced by H_2_/Pd/C to yield dihydrocinchonidine, and then reacted with 4-trifluoromethylbenzyl bromide to obtained **C14**. **C15** was prepared from cinchonidine via bromination, debromination and condensation with 4-trifluoromethylbenzyl bromide [14]. Quinine was reacted with 4-trifluoromethylbenzyl bromide or 3,5-bis(trifluoromethyl)benzyl bromide to obtain **C16** or **C17**.

In the beginning, the alkylation of **2** in the catalysis of **C1** in the two-phase system (toluene and 50% NaOH aqueous solution) was tested, although the yield was moderate (60.1%, entry 1 in Table 1), the enantiomeric ratio (**3a**:**3b**) was only 55:45. When the benzyl in **C1** was replaced by the bulky groups, such as methylnaphthalene or methylanthracene, neither the enantiomeric ratio was improved (Table 1, entry 2) nor the reaction took place (Table 1, entry 3). Subsequently, when the groups substituted at the *para*-position on the benzyl group were investigated, the structure–activity relationship showed that catalyst **C4** (with methyl substituent) did not work for the reaction (Table 1, entry 4) and those with Cl or F (**C5** and **C6**) worked well with an improvement in enantiocontrol (Table 1, entries 5 and 6). Fortunately, the *p*-CF_3_ derivative (**C7**) promoted the reaction to give a enantiomeric ratio of 83:17 (Table 1, entry 7). These findings suggested that the presence of electron-withdrawing groups on the benzyl group was favourable for the enantioselective reaction except the case of a nitro group (Table 1, entry 8). And then, the catalysts with a di-substituted benzyl group were examined. **C9** with 3.4-dichlorobenzyl resulted in a slightly higher enantiomeric ratio (68:32) than **C5** (Table 1, entry 9). But, neither **C10** nor **C11** (Table 1, entries 10 and 11) were as good as the mono-substituted counterparts (**C6** and **C7**). The derivatives (**C12**–**C15**) of **C7**, the best one so far, were further studied. When the hydroxy group in **C7** was protected by an allyl or a propargyl group, racemic product was obtained (Table 1, entries 12 and 13). This suggested that the free hydroxy group in **C7** was crucial to guarantee the stereoselectivity. Meanwhile, the good catalysis was maintained with both dihydrocinchonidine-derived **C14** and dehydro compound **C15**. Finally, the quaternary ammonium group from quinine was examined (Table 1, entries 16 and 17), and **C16** and **C17** gave the result inferior to the cinchonidine derivatives (**C7** and **C11**).

**Table 1:** Screening of phase-transfer catalysts for the asymmetry alkylation of **2**^a^.

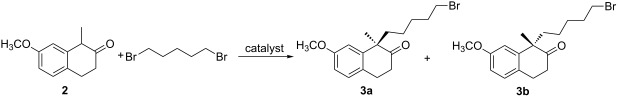			

entry	catalyst	yield^b^	**3a**:**3b**^c^

1	**C1**	60.1%	55:45
2	**C2**	58.3%	52:48
3	**C3**	no reaction	–
4	**C4**	no reaction	–
5	**C5**	47.5%	60:40
6	**C6**	65.3%	63:37
7	**C7**	62.0%	83:17
8	**C8**	no reaction	–
9	**C9**	58.7%	68:32
10	**C10**	43.4%	55:45
11	**C11**	52.2%	74:26
12	**C12**	65.0%	50:50
13	**C13**	65.0%	50:50
14	**C14**	63.5%	80:20
15	**C15**	61.1%	78:22
16	**C16**	50.3%	51:49
17	**C17**	53.2%	64:36

^a^The reaction was performed with 0.045 mol/L of **2** in toluene (24 mL), 3.0 equiv of 1.5-dibromopentane and 50% aq NaOH (2.4 mL) in the presence of 10 mol % of catalyst at 15–25 °C for 48 h under N_2_. ^b^Isolated yield including **3a** and **3b**. ^c^The enantiomeric ratio was determined by HPLC using a chiral column (Daicel chiral AY-H) with hexane/isopropyl alcohol 90:10 as the eluent, detected at 280 nm.

After a suitable catalyst (**C7**) was identified, further reaction optimization was performed (Table 2). In general, dichloromethane (DCM) was the common solvent for the two-phase reaction, but to our surprise, when the reaction was run in DCM (entry 2 in Table 2), it resulted in the racemic product. When other solvents, such as benzene, bromobenzene and fluorobenzene, were used, neither the enantiomeric ratio nor the yield was improved, compared with toluene as the solvent (Table 2, entries 1, 3–5). But, the reaction in chlorobenzene gave a slightly improved yield at a substrate concentration of 0.045 mol/L (Table 2, entry 1 and 6). Surprisingly, when the concentration increased to 0.07 mol/L, the improvement became more significant (Table 2, entries 7 and 8). However, further increasing the substrate concentration (Table 2, entry 9) decreased the stereoselectivity. For the screening of the base, the reduction of volume or concentration of 50% aq NaOH resulted in a decreased yield (Table 2, entries 11 and 12). If NaOH was replaced by K_2_CO_3_, no reaction took place (Table 2, entry 13). As far as the reaction temperature was concerned (Table 2, entry 7, 14 and 15), it was found that the reaction at 15–25 °C gave the best result. Finally, the reaction was scaled up (90 g of **2**) according to the conditions in entry 7, a similar outcome was obtained (Table 2, entry 16).

**Table 2:** Screening of catalytic conditions.

entry	solvent	concentration (mol/L)^a^	temperature (°C)	base^b^	yield^c^	**3a**:**3b**^d^

1	PhMe	0.045	15–25	50% aq NaOH	62.0%	83:17
2	CH_2_Cl_2_	0.045	15–25	50% aq NaOH	58.1%	50:50
3	PhH	0.045	15–25	50% aq NaOH	60.0%	81:19
4	PhBr	0.045	15–25	50% aq NaOH	58.9%	76:24
5	PhF	0.045	15–25	50% aq NaOH	60.4%	72:28
6	PhCl	0.045	15–25	50% aq NaOH	67.1%	81:19
7	PhCl	0.070	15–25	50% aq NaOH	76.2%	79:21
8	PhMe	0.070	15–25	50% aq NaOH	61.2%	77:23
9	PhCl	0.175	15–25	50% aq NaOH	70.8%	69:31
11^e^	PhCl	0.070	15–25	50% aq NaOH	48.1%	75:25
12	PhCl	0.070	15–25	25% aq NaOH	42.8%	72:28
13	PhCl	0.070	15–25	50% aq K_2_CO_3_	no reaction	–
14	PhCl	0.070	0–5	50% aq NaOH	incomplete	–
15	PhCl	0.070	35–40	50% aq NaOH	68.0%	75:25
16^f^	PhCl	0.070	15–25	50% aq NaOH	77.8%	79:21

^a^Concentration of compound **2** (5 g). ^b^The volume ratio of aqueous solution and organic solvent was 1:10. ^c^Isolated yield including **3a** and **3b**. ^d^The enantiomeric ratio was determined by HPLC using a chiral column (Daicel chiral AY-H) with hexane/isopropyl alcohol 90:10 as the eluent, detected at 280 nm. ^e^The volume of 50% aq NaOH was decreased to 5% of the volume of PhCl. ^f^90 g of **2** was added.

**Scheme 3:**
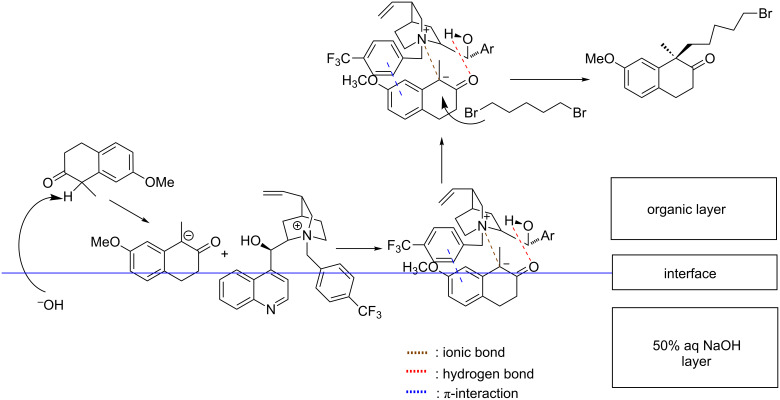
The proposed catalytic mechanism of stereoselective alkylation.

On the base of the above experimental results, a catalytic mechanism was proposed (Scheme 3). Compound **2** is deprotonated by sodium hydroxide into an anion in the organic layer. The anion goes to the interface between chlorobenzene and water, where it interacts with the quaternary ammonium group of catalyst **C7**. The distance between two molecules is getting close by the attraction between charges, then two additional interaction forces in the complex are produced on the same plane, including: 1) the carbonyl of **2** makes a hydrogen bond with the hydroxy group of **C7**; 2) the phenyl group of **2** forms a face-to-face π-stacking interaction with the benzyl moiety of **C7**. The complex of **2** with **C7** goes to the organic phase. Due to the sterical hindrance from the benzyl group, the alkylation by 1,5-dibromopentane takes place at the opposite side of the benzyl group of **C7** to afford **3a**.

## Conclusion

In summary, an enantioselective synthesis of (*R*)-(+)-1-(5-bromopentyl)-1-methyl-7-methoxy-2-tetralone (**3a**), a key intermediate of dezocine, in the catalysis of the quaternary ammonium benzyl bromides from cinchonidine was investigated and the best catalyst (**C7**) was identified. In addition, the preparation of **3a** with the optimized conditions was performed and the product was isolated in 77.8% yield with an enantiomeric ratio of 79:21. This method can be easily performed in large scale. In addition, the structure–activity relationships for the cinchona alkaloids catalysts were elucidated.

## Experimental

All solvents and reagents were of commercial sources and used without further purification. Melting points were determined on a Büchi Melting Point M-565 apparatus and are uncorrected. ^1^H and ^13^C NMR spectra were recorded using a Bruker 400 MHz spectrometer with TMS as an internal standard. Mass spectra were recorded with a Q-TOF mass spectrometer using electrospray positive ionization (ESI^+^). The enantiomeric ratio was determined by HPLC using a chiral column (Daicel chiral AY-H) with (hexane/isopropyl alcohol 90:10) as eluents, detected at 280 nm. Specific rotations were determined on a Rudolph Research Analytical automatic polarimeter IV. All reactions were monitored by TLC, which were carried out on silica gel GF254. Column chromatography was carried out on silica gel (200–300 mesh) purchased from Qindao Ocean Chemical Company of China.

### General procedure for the preparation of (*R*)*-*(+)-1-(5-bromopentyl)-1-methyl-7-methoxy-2-tetralone (**3a**)

To a stirred mixture of **2** (90.0 g, 0.47 mol), **C7** (25.2 g, 0.047 mol) and 1,5-dibromopentane (326.3 g, 1.4 mol) in chlorobenzene (6750 mL) was added 50% aq NaOH solution (675 mL) at 0 °C. The mixture was allowed to warm up slowly to 15–25 °C and stirred for 48 h under N_2_, and then aqueous layer was separated and extracted with chlorobenzene (700 mL). The combined organic layers were washed with 1 M HCl aqueous solution (2 L) and water (2 L), then the solvent and excess of 1,5-dibromopentane were recovered, respectively, under reduced pressure and then in vacuo. The residue was purified through a pad of silica gel (petroleum ether/EtOAc 10:1) to afford a colorless oil (125 g, 77.8%); Chiral purity (HPLC): **3a**/**3b** 79:21; ^1^H NMR (400 MHz, CDCl_3_) δ 7.09–7.07 (d, *J* = 8.0 Hz, 1H), 6.80 (s, 1H), 6.75–6.73 (d, *J* = 8.0 Hz, 1H), 3.81 (s, 3H), 3.29 (t, 2H), 2.99–2.96 (m, 2H), 2.67–2.54 (m, 2H), 2.11–2.07 (m, 1H), 1.73–1.63 (m, 3H), 1.38 (s, 3H), 1.32–1.29 (m, 2H), 1.04–0.86 (m, 2H); ^13^C NMR (100 MHz, CDCl_3_/TMS) δ 214.55,158.74, 143.40, 129.00, 128.16, 112.44, 111.33, 55.32, 51.80, 40.13, 38.57, 33.71, 32.42, 28.47, 27.60, 27.35, 24.28; MS (ESI^+^) *m*/*z*: 339.00 [M + H]^+^.

The above-obtained product underwent subsequent cyclization, oximation and reduction according to the literature [10] (without resolution) to get compound **6a**, and then **6a** was transformed to dezocine with 23.0% overall yield and 100% purity. The mp, optical rotation value, MS and ^1^H NMR of the product were consistent with those in the literature [4,10].

## Supporting Information

File 1 Synthesis of catalysts **C1**–**C17**, synthesis of dezocine, ^1^H NMR and MS spectra of catalysts **C1–C17** and chiral HPLC diagrams of **3**. ^1^H NMR, ^13^C NMR, MS spectra of **3**. ^1^H NMR, MS spectra HPLC diagrams of dezocine.
