# Supplementary material for: Enantioselective phase-transfer catalyzed alkylation of 1-methyl-7-methoxy-2-tetralone: an effective route to dezocine
Source: Beilstein J Org Chem. 2018 Jun 11;14:1421–7. doi: 10.3762/bjoc.14.119 (PMC6009193; doi:10.3762/bjoc.14.119)
Supplement: File 1 — Synthesis of catalysts C1–C17, synthesis of dezocine, 1H NMR and MS spectra of catalysts C1–C17 and chiral HPLC diagrams of 3. 1H NMR, 13C NMR, MS spectra of 3. 1H NMR, MS spectra HPLC diagrams of dezocine. [file Beilstein_J_Org_Chem-14-1421-s001.pdf]

Supporting Information

for

Enantioselective phase-transfer catalyzed alkylation of 1-methyl-7-methoxy-2-tetralone: an effective route to dezocine

Ruipeng Li, Zhenren Liu, Liang Chen, Jing Pan, and Weicheng Zhou*

Address: State Key Lab of New Drug & Pharmaceutical Process, Shanghai Key Lab of Anti-Infectives, Shanghai Institute of Pharmaceutical Industry, China State Institute of Pharmaceutical Industry, No. 285, Gebaini Rd., Shanghai 201203, P. R. of China

Email: Weicheng Zhou* - zhouweicheng58@163.com

*Corresponding author

Synthesis of catalysts C1–C17, synthesis of dezocine, ^{1}H NMR and MS spectra of catalysts C1–C17 and chiral HPLC diagrams of 3. ^{1}H NMR, ^{13}C NMR, MS spectra of 3. ^{1}H NMR, MS spectra HPLC diagrams of dezocine

Table of contents

1. Synthesis of catalysts **C1**–**C17**.

2. Synthesis of dezocine

3. ^{1}H NMR and MS spectra of catalyst **C1**–**C17** and chiral HPLC diagrams of **3**.

4. ^{1}H NMR, ^{13}C NMR, MS spectra of **3**.

5. ^{1}H NMR, MS spectra HPLC diagrams of dezocine

1. **Synthesis of catalysts C1–C17**.

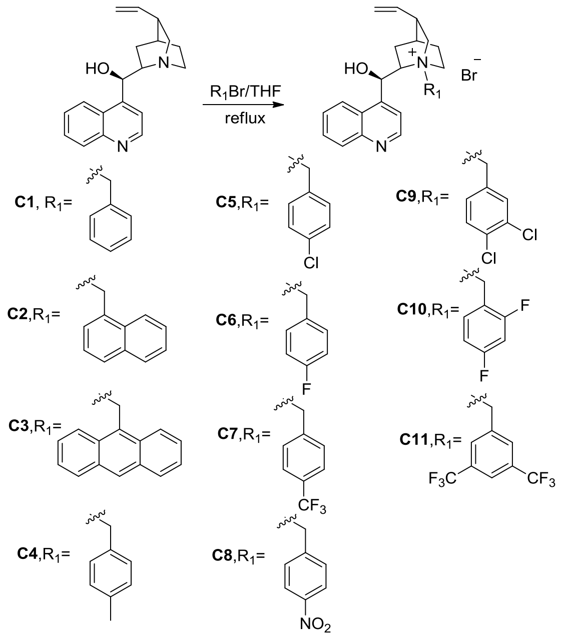

The phase-transfer catalysts (**C1**–**C11**) were synthesized according to the procedures below. To a solution of cinchonidine (1.00 g, 3.4 mmol) in THF (50 mL) was added the aryl benzyl bromides (3.4 mmol). The mixture was heated for 6–8 h at reflux. After cooling to room temperature, the mixture was poured into MTBE (150 mL) under stirring. The precipitated solid was filtrated and recrystallized from CH_3OH/MTBE to afford **C1**–**C11**.

C1, white solid (yield: 88%); mp:190 °C (dec.); $[\alpha]_D^{13}$=-179° (*c*=0.1, CH_3OH); ^1H NMR (400 MHz, DMSO-*d6*) δ: 8.90 (d, *J* = 4.0Hz, 1H), 8.31-8.33 (d, *J* = 8.0Hz, 1H), 8.11-8.13 (d, 1H), 7.96-8.02 (q, *J* = 16.0 Hz, 4H), 7.82-7.88 (m, 2H), 7.76-7.78 (m, 4H), 6.56-6.76 (m, 2H), 5.68 (m, 1H), 5.27-5.30 (d, *J* = 12.0Hz, 1H), 5.14-5.19 (m, 2H), 4.95-4.98 (d, *J* = 12.0Hz, 1H), 4.32 (t, 1H), 3.96 (t, 1H), 3.82 (m, 1H), 3.23-3.37 (m, 3H), 2.00-2.17 (m, 3H), 1.79-1.84 (m, 1H), 1.24-1.33 (m, 1H). MS (ES^+) m/z: 385.10 $[M]^+$.

C2, white solid (yield: 80%); mp:220 °C (dec.); $[\alpha]_D^{13}$=-260° (c=0.1, CH_3OH); ^1H NMR (400 MHz, DMSO-*d6*) δ:9.03-9.02 (d, *J* = 4.0Hz, 1H), 8.22-8.13 (dd, *J* = 8.0Hz, 2H), 8.11-8.13 (d, 1H), 7.79-7.88 (t, 2H), 7.76-7.77 (d, 1H), 7.74-7.75 (m, 2H), 7.62-7.74 (m, 4H), 6.94-6.95 (d, 1H), 6.77 (m, 1H), 5.68-5.82 (m, 2H), 5.37-5.39 (d, 1H), 5.11-5.15 (d, 1H), 4.96-4.98 (d, 1H), 4.42 (t, 1H), 4.23 (t, 1H), 3.88-3.91 (m, 1H),3.31-3.37(m, 1H),3.10 (m,1H), 2.61 (s, 1H), 1.97-2.21 (m, 3H), 1.72 (t, 1H), 1.33 (m, 1H); MS (ES^+) m/z:435.24 $[M]^+$.

C3, yellow solid (yield: 78%); mp: 158 °C (dec.); $[\alpha]_D^{13}$=-337° (c=0.1, CH_3OH); ^1H NMR (400 MHz, DMSO-*d6*) δ: 8.98-9.00 (d, *J* = 8.0 Hz, 1H), 8.77-8.82 (m, 2H), 8.60-8.62 (d, *J* = 8.0 Hz 1H), 8.15-8.17 (d, *J* = 8.0 Hz, 1H), 8.00-8.01 (d, 1H), 7.90 (s,1H), 7.63-7.65 (d, 1H), 7.49-7.56 (m, 2H), 7.28-7.32 (m, 1H),7.15-7.18 (m, 4H), 7.10-7.14 (m, 2H), 6.76-6.79 (d, *J* = 12.0 Hz 1H), 6.55-6.58 (d, *J* = 12.0 Hz 1H), 5.31-5.35 (m, 1H), 5.17-5.22 (d, 1H), 4.68 (m, 2H), 4.00 (d, 1H), 2.51-2.55 (t, 1H), 2.33-2.49 (t, 1H), 2.06 (s, 1H), 1.70-1.74 (m, 2H), 1.20 (m, 1H), 1.02 (t, 1H), 0.82 (m, 1H). MS (ES^+) m/z: 485.26 $[M]^+$.

C4, white solid (yield: 90%); mp: 228–230 °C (dec.); $[\alpha]_D^{15}$=-195° (c=0.1, CH_3OH); ^1H NMR (400 MHz, DMSO-d6) δ: 8.96 (d, *J* = 4.0 Hz, 1H), 8.28 (d, *J* = 8.0 Hz, 1H), 8.10 (d, *J* = 4.0 Hz, 1H), 7.80-7.85 (m, 3H), 7.72-7.75 (t, 1H), 7.60 ‐ 7.61 (m, *J* = 8Hz 2H), 7.36-7.38 (d, *J* = 8 Hz, 2H), 6.71(d, *J* = 4 Hz, 1H), 6.53 (s, 1H), 5.62-5.70 (m, 1H), 5.12 (m, 2H), 4.95 (t, 2H), 4.25 (t, *J* = 20.0 Hz, 2H), 3.90 (t, 1H), 3.71 (dd, *J* = 4.0 Hz, 1H), 3.17-3.29 (m, 2H), 2.67 (s, 1H), 2.38 (s, 3H), 2.12-1.97 (m, 3H), 1.80 (t, 1H), 1.22–1.30 (m, 1H). MS (ES^+) m/z: 399.29 $[M]^+$.

C5, white solid (yield: 87%); mp: 240–242℃ (dec.); $[\alpha]_D^{15}$=-180° (c=0.1, CH_3OH); ^{1}H NMR (400 MHz, DMSO-*d6*)δ: 8.97 (d, *J* = 4.0 Hz, 1H), 8.27 (d, *J* = 8.0Hz, 1H), 8.01 (d, *J* = 8.0Hz, 1H), 7.86-7.72 (m, 5H), 7.65-7.62 (d, 2H), 6.71-6.70 (d, *J* = 4.0Hz 1H), 6.52 (d, 1H), 5.70-5.62 (m, 1H), 5.16-5.12 (m, 2H), 5.02-4.92 (m, 2H), 4.23 (t, 1H), 3.89 (t, 1H), 3.73 (m, 1H), 3.25-3.18 (m,1H), 2.62 (s, 1H), 2.14-1.98 (m, 3H), 1.79 (bs, 1H), 1.30-1.22 (m, 2H); MS (ES^+) m/z: 419.30 $[M]^+$.

C6, white solid (yield: 93%); mp: 225 °C (dec.); $[\alpha]_D^{15}$=-187° (c=0.1, CH_3OH); ^{1}H NMR (400 MHz, DMSO-*d6*) δ : 8.99 (d, *J* = 4.0 Hz, 1H), 8.29 (d, *J* = 8.0 Hz, 1H), 8.12 (d, *J* = 8.0 Hz, 1H), 7.88-7.72 (m, 5H), 7.46-7.41 (d, 2H), 6.73-6.72 (d, *J* = 4.0 Hz, 1H), 6.55 (d, 1H), 5.73-5.64 (m, 1H), 5.19-5.14 (m, 2H), 5.04-4.95 (m, 2H), 4.26 (t, 1H), 3.91 (t, 1H), 3.75-3.70 (m, 1H), 3.35-3.29 (m, 1H), 3.27-3.20 (s, 1H), 2.68 (s, 1H) 2.16-2.01 (m, 3H), 1.85-1.79 (t, 1H), 1.33-1.24 (m, 2H); MS (ES^+) m/z: 403.37 $[M]^+$.

C7, white solid (yield:85%); mp: 222–223 °C; $[\alpha]_D^{25}$=-159° (c=0.1 CH_3OH); ^{1}H NMR (400 MHz, DMSO-*d6*) δ: 8.99 (d, *J*= 4.0 Hz, 1H), 8.32 (d, *J*=8.0Hz, 1H), 8.13-8.11 (d, *J*=8.0 Hz, 1H), 8.02-7.96 (m, 4H), 7.88-7.82 (m, 2H), 7.78-7.76 (m, 1H), 6.77-6.76 (d, *J* = 4.0 Hz, 1H), 6.57-6.56 (d, *J* = 4.0 Hz, 1H), 5.73-5.64 (m, 1H), 5.30–5.27 (m, 1H), 5.19-5.14 (m, 2H), 4.32 (t, 1H), 3.98 – 3.94 (t, 1H), 3.82-3.81 (m, 1H), 3.37–3.23 (m, 3H), 2.68 (s, 1H), 2.17 – 2.00 (m, 3H), 1.82 (t, 1H), 1.33–1.24 (m, 1H); MS (ES^+) m/z: 453.14 $[M]^+$.

C8, white solid (yield: 95%); mp:195 °C (dec.); $[\alpha]_D^{15}$=-175° (c=0.1, CH_3OH); ^{1}H NMR (400 MHz, DMSO-*d6*) *δ*: 8.99 (d, *J*=8.0Hz, 1H), 8.45-8.42 (d, *J* = 12.0 Hz, 2H),

8.33-8.30 (d, *J* = 8.0 Hz 1H), 8.14-8.05 (m, 3H), 7.88-7.75 (m, 3H), 6.76-6.75 (d, *J* =4.0Hz, 1H), 6.57-6.56 (d, *J*=4.0Hz 1H), 5.73-5.65 (m, 1H), 5.32-5.29 (d,1H), 5.20-5.15 (m, 2H), 4.98-4.96 (m, *J* = 8.0 Hz, 1H), 4.32 (t, 1H), 3.96 (t, 2H), 3.83-3.78 (m, 1H), 3.37-3.29 (m, 1H), 2.27-3.23 (m, 1H), 2.65 (s, 1H), 2.18-2.01 (m, 3H), 1.83-1.77 (t, 1H), 1.34-1.24 (m, 2H); MS (ES^+) m/z: 430.22 $[M]^+$.

C9, white solid (yield:87%); mp:237–240 °C (dec.); $[\alpha]_D^{15}$=-175° (c=0.1, CH_3OH); ^1H NMR (400 MHz, DMSO-*d6*) δ: 8.98 (d, *J* = 4.0 Hz, 1H), 8.30 (d, *J* = 8.0 Hz, 1H), 8.12-8.10 (m, 2H), 7.88-7.73 (m, 5H), 6.71-6.70 (d, *J* = 4.0 Hz, 1H), 6.52 (d, 1H), 5.72-5.63 (m, 1H), 5.22-5.16 (m, 2H), 5.10-4.94 (m, 2H), 4.29 (t, 1H), 3.90 (t, 1H), 3.77 (m, 1H), 3.42-3.36 (m, 1H), 3.29-3.25 (s, 1H), 2.26 (s, 1H) 2.15-2.00 (m, 3H), 1.81 (bs, 1H), 1.31-1.24 (m, 2H); MS (ES^+) m/z: 453.15 $[M]^+$.

C10, white solid (yield: 83%); mp: 205 °C (dec.); $[\alpha]_D^{15}$=-163° (c=0.1, CH_3OH); ^1H NMR (400 MHz, DMSO-*d6*) δ : 8.99 (d, *J* = 4.0 Hz, 1H), 8.33 (d, *J* = 8.0 Hz, 1H), 8.13-8.11 (m, 2H), 7.98-7.99 (q, 1H), 7.86-7.76 (m, 3H), 7.57-7.52 (m, 1H), 7.39-7.35 (m, 1H), 6.79-6.78 (d, *J* = 4.0 Hz, 1H), 6.79-6.78 (d, *J* = 4.0 Hz 1H), 6.56 (s, 1H), 5.74-5.66 (m, 1H), 5.26-5.16 (m, 2H), 5.04-4.96 (m, 2H), 4.27 (t, 1H), 4.02 (t, 1H), 3.77 (m, 1H), 3.46-3.41 (m, 1H), 3.24-3.17 (s, 1H), 2.69 (s, 1H) 2.15-2.01 (m, 3H), 1.85-1.79 (t, 1H), 1.32-1.24 (m, 2H); MS (ES^+) m/z: 421.34 $[M]^+$.

C11, white solid (yield: 88%); mp: 228–231 °C (dec.); $[\alpha]_D^{15}$=-164° (c=0.1, CH_3OH); ^1H NMR (400 MHz, DMSO-*d6*) δ: 8.99(d, *J* = 8.0 Hz, 1H), 8.53 (s, 1H), 8.38 (s, 1H), 8.31-8.29 (d, *J* = 8.0 Hz, 1H), 8.14-8.12 (d, *J* = 8.0 Hz, 1H), 7.89-7.75 (m, 3H), 6.71-6.70 (d, *J* = 4.0 Hz, 1H), 6.56-6.54 (d, *J* = 8.0 Hz, 1H), 5.74-5.65 (m, 1H),

5.41-5.38 (d, 1H), 5.27-5.16 (m, 2H), 4.99-4.96 (m, J = 12.0 Hz, 1H), 4.40 (t, 1H), 3.92-3.82 (t, 2H), 3.43-3.37 (m, 1H), 3.31-3.25 (m, 1H), 2.65 (s, 1H), 2.20-2.03 (m, 3H), 1.83-1.78 (t, 1H), 1.36-1.34 (m, 2H); MS (ES^{+}) m/z: 521.12 [M]$^{+}$.

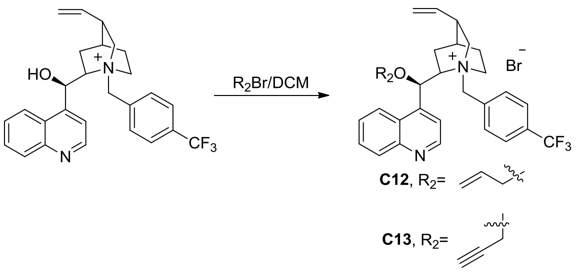

The phase-transfer catalysts (**C12** and **C13**) were synthesized according to the procedure below. To a solution of C7 (0.5 g) and propylene bromide or propargyl bromide (3 equiv) in dichloromethane (5 mL) was added 0.5 mL 50% KOH aqueous solution at rt. The mixture was stirred for 4 h and then quenched with water (5 mL). The aqueous layer was extracted with dichloromethane (2.5 mL). The collected organic layers were dried over Na_2SO_4, concentrated in vacuum and then purified by flash chromatography (dichloromethane/methanol 20:1) to afford the product **C12** and **C13**.

C12, white solid (yield: 85%); mp: 168–170°C (dec.); $[\alpha]_D^{15}$=-155° (c=0.1, CH_3OH); ^{1}H NMR (400 MHz, DMSO-d_6) δ: 8.98-8.97 (d, J = 4.0 Hz, 1H), 8.88-8.86 (d, J = 8.0 Hz, 1H), 8.15-8.13 (d, J = 8.0 Hz, 3H), 7.91–7.74 (m, 4H), 6.87-6.84 (d, 1H), 6.26 (s, 1H), 6.15-6.07 (m, 1H), 5.75–5.67 (m, 1H), 5.44-5.39 (m, 1H), 5.10–5.03 (q, 2H), 4.78-4.71 (m, 2H), 4.39 (t, 1H), 4.31-4.28 (d, 1H), 4.11-4.06 (m, 1H), 3.35 (t, 1H), 3.18 (t, 1H), 2.63 (s, 1H), 2.18-2.09 (m, 3H), 1.78 (m, 2H), 1.26 (s, 1H); MS (ES^{+}) m/z: 493.08 [M]$^{+}$.

C13,white solid (yield: 65%); mp:140 °C (dec.); $[\alpha]_D^{15}$=-168° (c=0.1, CH_3OH); ^{1}H

NMR (400 MHz, $CDCl_3$) *δ*: 8.98-8.97 (d, *J* = 4.0 Hz, 1H), 8.85-8.82 (d, *J* = 12.0 Hz, 1H), 8.21-8.15 (m, 3H), 8.02 (s, 1H), 7.85–7.77 (m, 3H), 7.50 (s, 1H), 6.89-6.86 (d, 1H), 6.49 (s, 1H), 5.78–5.70 (m, 1H), 5.43-5.39 (m, 1H), 5.30 (s, 1H), 5.10-5.05 (m, 2H), 4.85 (s, 1H), 4.62-4.57 (dd, 1H), 4.41-4.33 (d, 1H), 4.20-4.15 (m, 1H), 3.30 (t, 1H), 3.20 (t, 1H), 2.67-2.63 (m, 2H), 2.22-2.06 (m, 3H), 1.85 (m, 1H), 1.54 (s, 1H); MS (ES^+) m/z: 491.13 $[M]^+$.

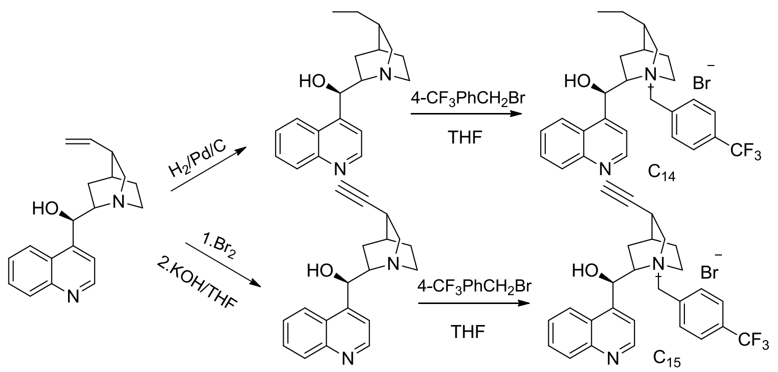

C14, 1) To a solution of cinchonidine (2.0 g, 6.80 mmol) in methanol (20 mL) was added 10% Pd/C (0.2 g) at 25 °C. The mixture was stirred for 3 h under H_2 atmosphere. After the hydrognation was finished, Pd/C was removed by filtration and the filtrate was concentrated in vacuum to afford the crude intermediate (2.0 g, 100%). 2) To the crude intermediate was added 4-trifluoromethylbenzyl bromide (1.68 g, 7.50 mmol) and tetrahydrofuran (40 mL) at room temperature. The reaction mixture was stirred for 6 h at reflux, concentrated in vacuum and then recrystallization from 2 mL methanol to afford a white solid (2.9 g, yield 81%). mp: 225 °C (dec.); $[\alpha]_D^{15}$=-138° (c=0.1, CH_3OH); ^1H NMR (400 MHz, DMSO-d_6) *δ*: 8.99 (d, *J* = 4.0 Hz, 1H), 8.30 (d, *J* = 8.0 Hz, 1H), 8.12-8.10 (d, *J* = 8.0 Hz, 1H), 7.95 (m, 4H), 7.87-7.81 (m, 2H), 7.76-7.73 (m, 1H), 6.73-6.72 (d, *J* = 4.0 Hz, 1H), 5.26-5.23 (d, *J* = 12.0 Hz, 1H), 5.03-5.00 (d, *J* = 12.0Hz, 1H), 4.29 (t, 1H), 3.93 (t, 1H), 3.48-3.46 (m, 1H), 3.28–3.17

(m, 2H), 2.15–1.95 (m, 3H), 1.78-1.69 (t, 2H), 1.37–1.31 (m, 1H), 1.23–1.10 (m, 2H), 0.71-0.68 (m, 1H); MS (ES^+) m/z: 455.28 $[M]^+$.

C15, 1)To a solution of cinchonidine (2.0 g, 6.80 mmol) in DCM (20 mL) was added dropwise a solution of bromine (2.17 g, 13.6 mmol) in DCM (2.5 mL) at 0 °C. The reaction mixture was stirred for 1h at room temperature and poured into petroleum ether (100 mL). The solid was collected and washed with petroleum ether (50 mL), then dried in vacuo. The solid was added to the suspension of KOH (0.76 g) and tetrabutylammonium bromide (0.2 g) in THF (20 mL). The mixture was stirred for 4 h at reflux and then cooled to rt, The product was collected and recrystallization from ethyl acetate (1.4 g, 71%). 2) To a solution of the above product (1.4 g) in THF (40 mL) was added 4-trifluoromethylbenzyl bromide, and it was stirred for 6 h at reflux and cooled to room temperature, and then poured into MTBE (120 mL) with stirring. The precipitated solids were isolated by filtration, and then recrystallized from MeOH/MTBE to afford **C15** (2.3 g, 92%). mp: 218–220 °C (dec.); $[\alpha]_D^{15}$=-264° (c=0.1, CH_3OH); ^{1}H NMR (400 MHz, DMSO-d_6) δ: 9.03 (d, J = 4.0 Hz, 1H), 8.36-8.33 (d, J = 12.0 Hz, 1H), 8.15-8.13 (d, J = 8.0 Hz, 1H), 8.02-7.98 (m, 4H), 7.90-7.77 (m, 2H), 6.84-6.83 (d, J = 4.0 Hz, 1H), 6.57-6.56 (d, J = 4.0 Hz, 1H), 5.20 (s, 2H), 4.33 (m, 1H), 4.12 (t, 2H), 3.95-3.92 (d, 1H), 3.39 (t, 1H), 3.24 (t, 1H), 3.03–3.00 (m, 2H), 2.25 (t, 1H), 2.16 (s, 1H), 2.03 (t, 1H), 1.76 (m, 1H), 1.46 (t, 1H); MS (ES^+) m/z: 451.23$[M]^+$.

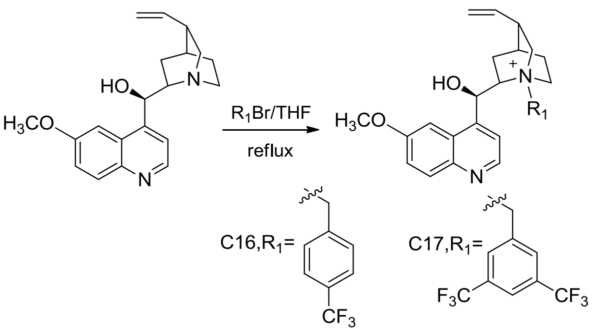

The phase-transfer catalysts (**C16**, **C17**) were synthesized according to the **C7** and **C11**.

C16,white solid (yield:76%); mp: 190–194 °C (dec.); $[\alpha]_D^{15}$=-198° (c=0.1, CH_3OH); ^{1}H NMR (400 MHz, DMSO-*d6*) δ: 8.83-8.82 (d, *J* = 4.0 Hz, 1H), 8.05-8.02 (d, *J* = 12.0 Hz, 1H), 7.96 (m, 4H), 7.78 – 7.77 (d, *J* = 4.0Hz, 1H), 7.53-7.50 (q, 1H), 7.40 (d, 2H), 6.72-6.71 (d, *J* = 4Hz, 1H), 6.59-6.58 (t, *J* = 4.0Hz, 1H), 5.80 – 5.71 (m, 1H), 5.55-5.52 (d, 1H), 5.14–5.00 (q, 1H), 4.85–4.82 (d, 1H), 4.03 (s, 3H), 3.89 (t, 1H), 3.76-3.71 (m, 1H), 2.72–2.66 (m, 1H), 2.28-2.12 (m, 2H), 2.02 (s, 1H), 1.83 (t, 1H), 1.47 (t, 1H); MS (ES^+) m/z: 483.16 $[M]^+$.

C17,white solid (yield: 79%); mp: 198–200 °C (dec.); $[\alpha]_D^{15}$=-196° (c=0.1, CH_3OH); ^{1}H NMR (400 MHz, DMSO-*d6*) δ: 8.84-8.82 (d, *J* = 8.0 Hz, 1H), 8.46-8.38 (m, 3H), 8.06-8.04 (d, *J* = 8.0 Hz, 1H), 7.76 – 7.75 (d, *J* = 4.0 Hz, 1H), 7.55-7.54 (d, 1H), 7.39-7.38 (d, 1H), 6.69-6.68 (d, *J* = 4Hz, 1H), 6.52 (s, 1H), 5.81 – 5.72 (m, 1H), 5.56-5.53 (d, 1H), 5.15–5.11 (q, 1H), 5.04–4.93 (q, 2H), 4.34(t, 1H), 4.02(s, 1H), 3.83-3.71 (m, 2H), 3.48–3.42 (m, 1H), 3.30-3.24 (m, 1H), 2.68-2.63 (m, 1H), 2.29-2.24 (m, 1H), 2.19-2.12 (m, 1H), 2.03-2.02 (d, 1H), 1.85-1.77 (m, 1H), 1.49 (t, 1H); MS (ES^+) m/z: 551.21 $[M]^+$.

2. Synthesis of dezocine

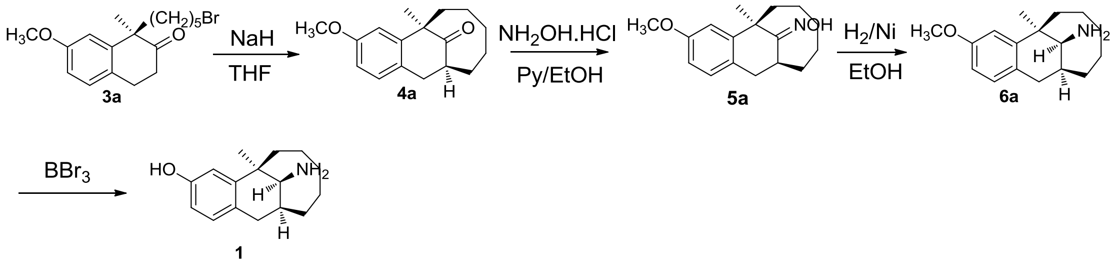

The compound **3a** (contaminated with 21% 3b) underwent subsequent cyclization, oximation and reduction according to the literature [10] (without resolution) to get compound **6a** (yield: 38%), The hydrochloride of **6a** showed $[\alpha]_D^{20}$= -50° (*c*=2.8, MeOH), Ref. 10 $[\alpha]_D^{20}$= -50.5° (*c*=2.8, MeOH); ^{1}H NMR (400 MHz, $CDCl_3$) *δ*: 8.86 (s, 3H), 7.05 (d, *J* = 8.0 Hz, 1H). 6.76-6.73 (M, 1H), 3.80 (s, 3H), 3.73 (m, 1H), 3.24-3.18 (m, 1H), 2.86-2.71 (m, 2H). 2.19-2.04 (m, 2H), 1.93-1.89 (m, 2H) 1.66-1.53 (m, 7H), 0.94-0.77 (m, 2H). MS (ES+) m/z: 260.13 [M+H].

6a was demethylated according to the literature [10] to dezocine (overall yield: 23.0%, based on **3a**); Chiral purity (HPLC):100%; chemical purity (HPLC):100%; $[\alpha]_D^{rt}$= -52° (*c*=1, MeOH); mp: 168℃; ^{1}H NMR (400 MHz, $CDCl_3$) *δ*:8.91 (s, 1H), 6.84 (d, *J* = 8.0 Hz, 1H), 6.61 (d, 1H), 6.52-6.49 (dd, *J* = 4.0 and 8.0, 1H,), 3.00-2.94 (m, 2H), 2.53-2.49 (m, 1H), 2.08-2.02 (m, 2H), 1.76-1.72 (m, 1H), 1.56-1.40 (m, 7H), 1.25-1.08 (m, 1H), 0.80-0.72 (m, 2H). MS (ES+) m/z: 246.08 [M+H].

3. ^{1}H NMR and MS spectra of catalyst C1–C17 and chiral HPLC diagrams of 3.

^{1}H NMR and MS spectra of catalyst C1.

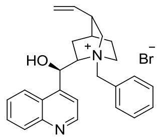

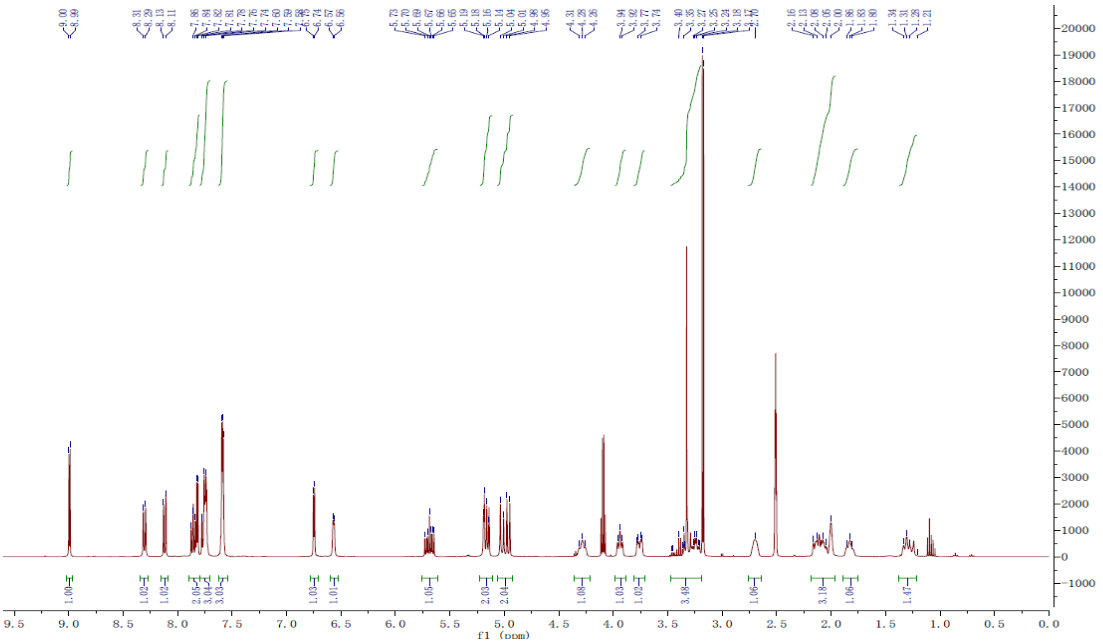

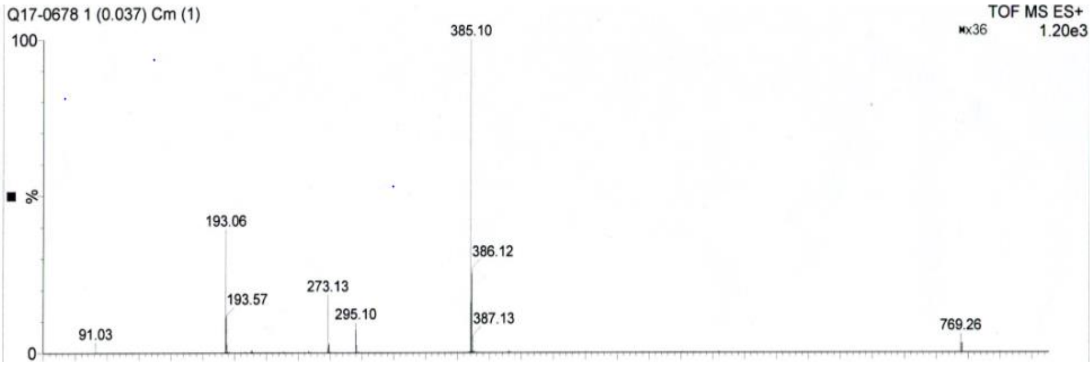

Chiral HPLC diagrams of 3 in catalysis of C1

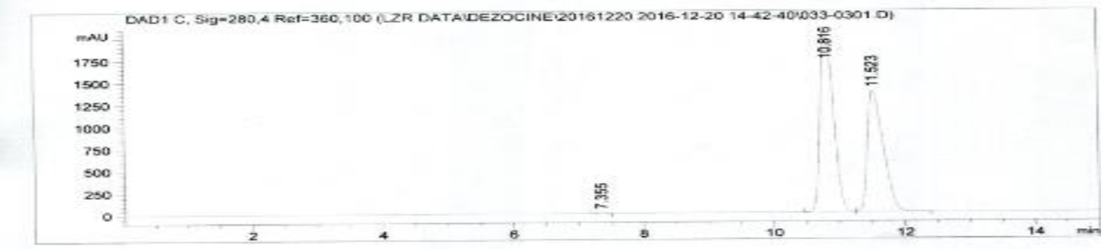

Area Percent Report

Sorted By : Signal
Multiplier : 1.0000
Dilution : 1.0000
Use Multiplier & Dilution Factor with ISTDs

Signal 1: DAD1 C, Sig=280,4 Ref=360,100

Peak #	RetTime [min]	Type	Width [min]	Area [mAU*s]	Height [mAU]	Area %
1	7.355	VV	0.1168	133.30516	17.52336	0.2550
2	10.816	VV	0.2330	2.89674e4	2000.10669	55.4076
3	11.523	VV	0.2578	2.31798e4	1381.32141	44.3374

^{1}H NMR and MS spectra of catalyst C2.

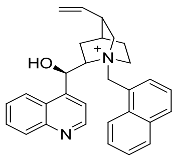

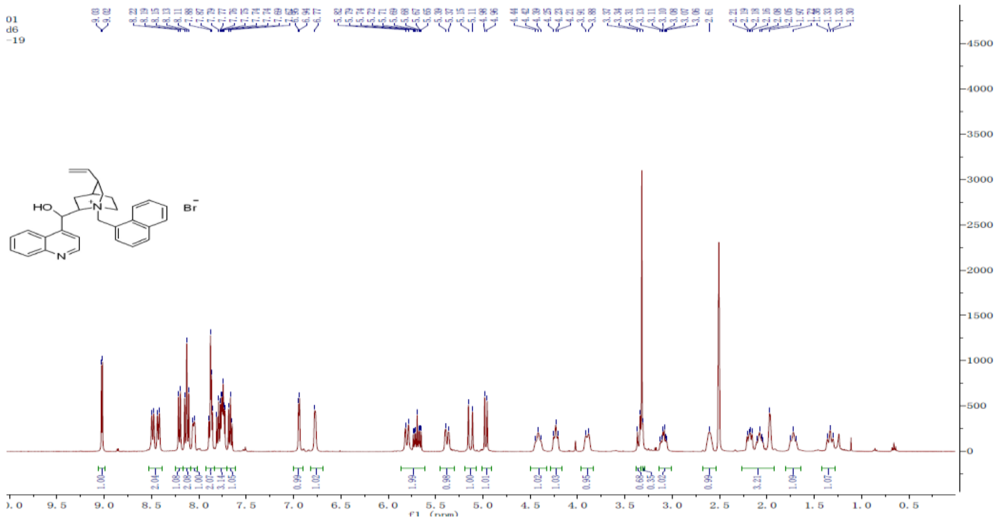

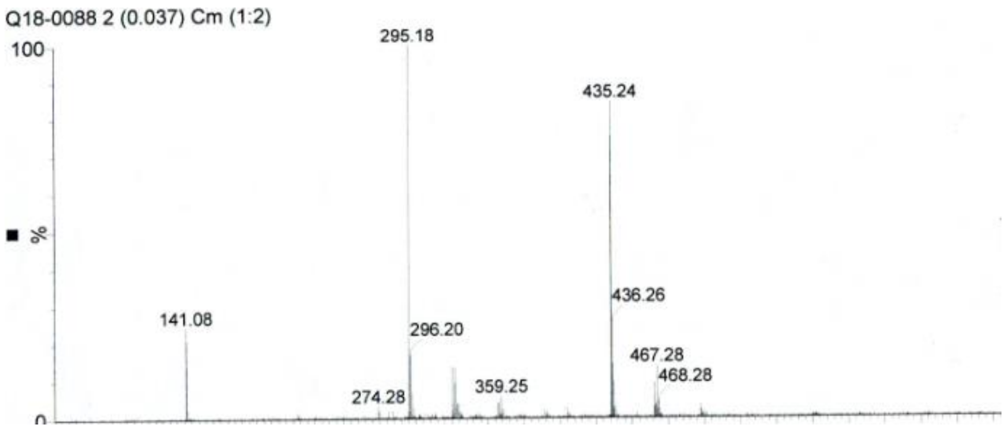

Chiral HPLC diagrams of 3 in catalysis of C2

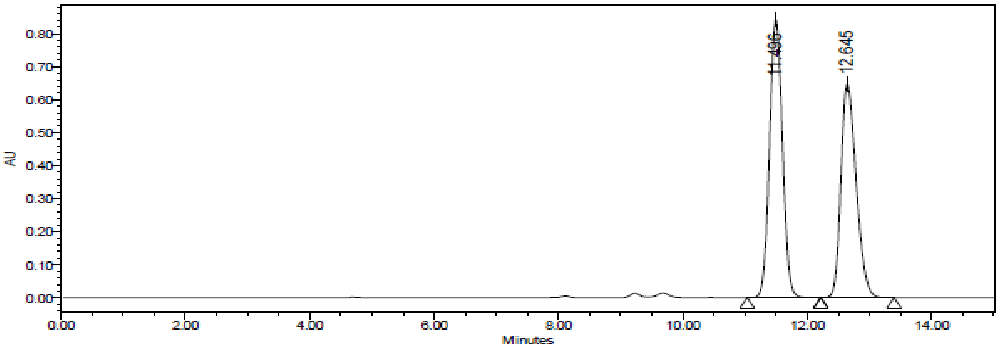

Peak Results

	Name	RT	Area	Height	Amount	Units
1		11.496	12152423	841966		
2		12.645	10998894	647309		

^{1}H NMR and MS spectra of catalyst C3.

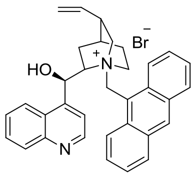

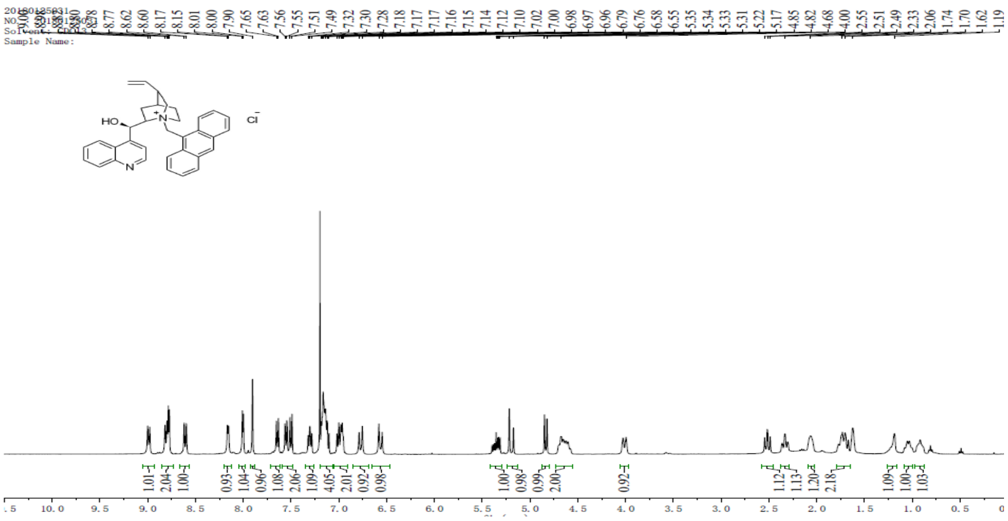

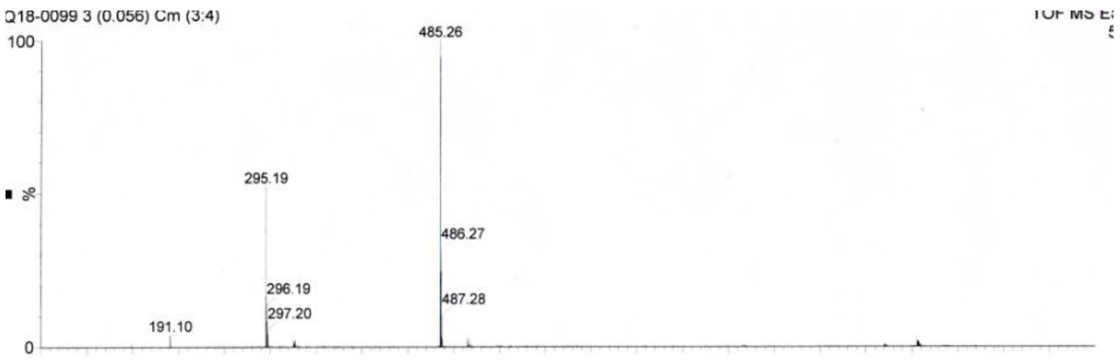

^{1}H NMR and MS spectra of catalyst C4.

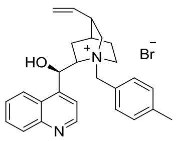

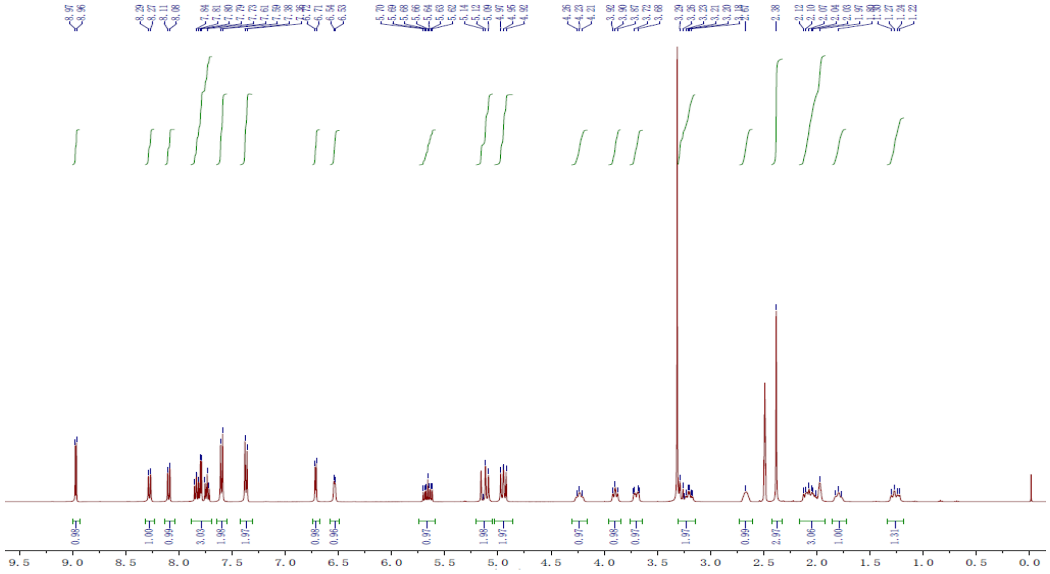

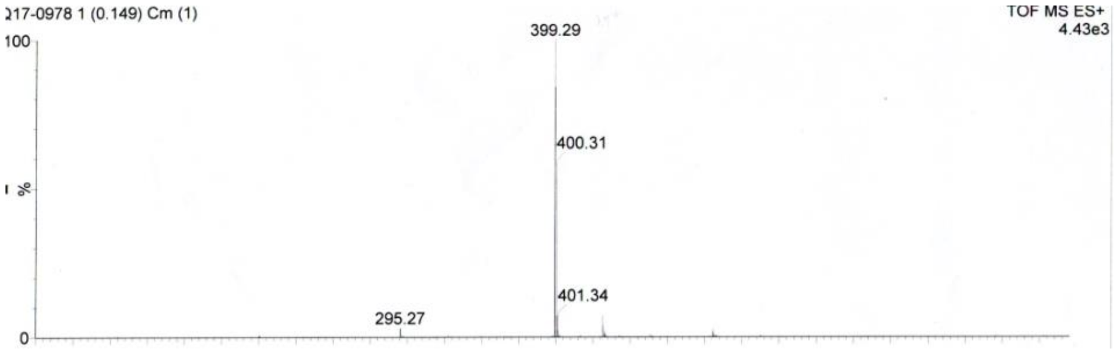

^{1}H NMR and MS spectra of catalyst C5.

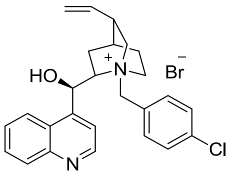

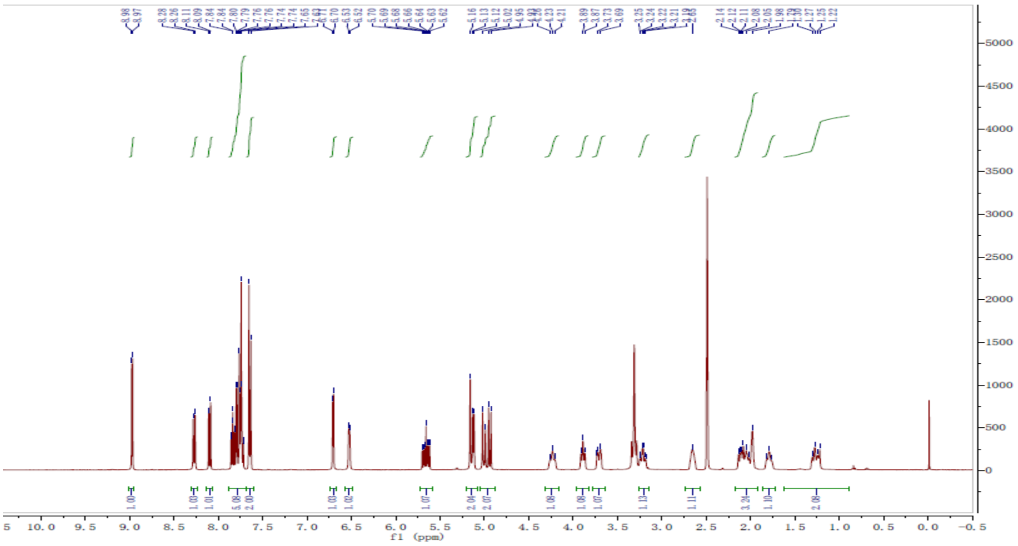

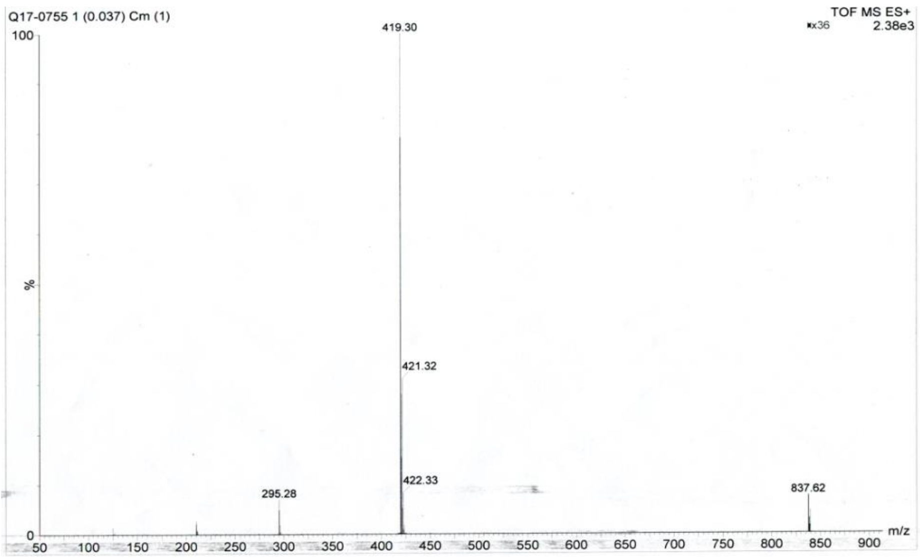

Chiral HPLC diagrams of 3 in catalysis of C5

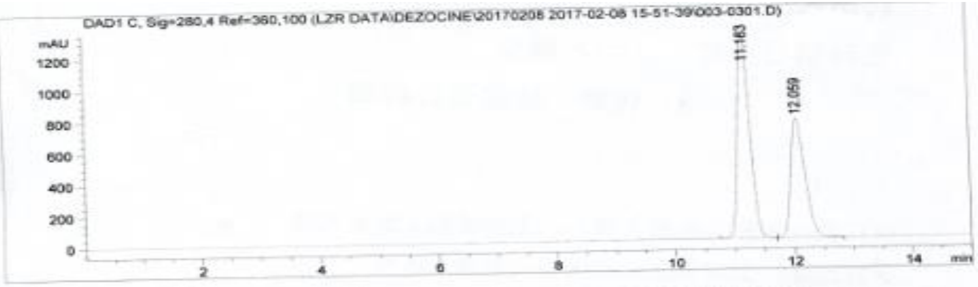

Area Percent Report

Sorted By : Signal
Multiplier : 1.0000
Dilution : 1.0000
Use Multiplier & Dilution Factor with ISTDs

Signal 1: DAD1 C, Sig=280,4 Ref=360,100

Peak #	RetTime [min]	Type	Width [min]	Area [mAU*s]	Height [mAU]	Area %
1	11.183	VV	0.2248	1.85868e4	1299.71729	59.6436
2	12.059	VV	0.2542	1.25763e4	763.30341	40.3564

^{1}H NMR and MS spectra of catalyst C6.

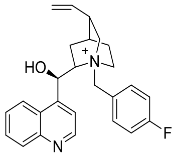

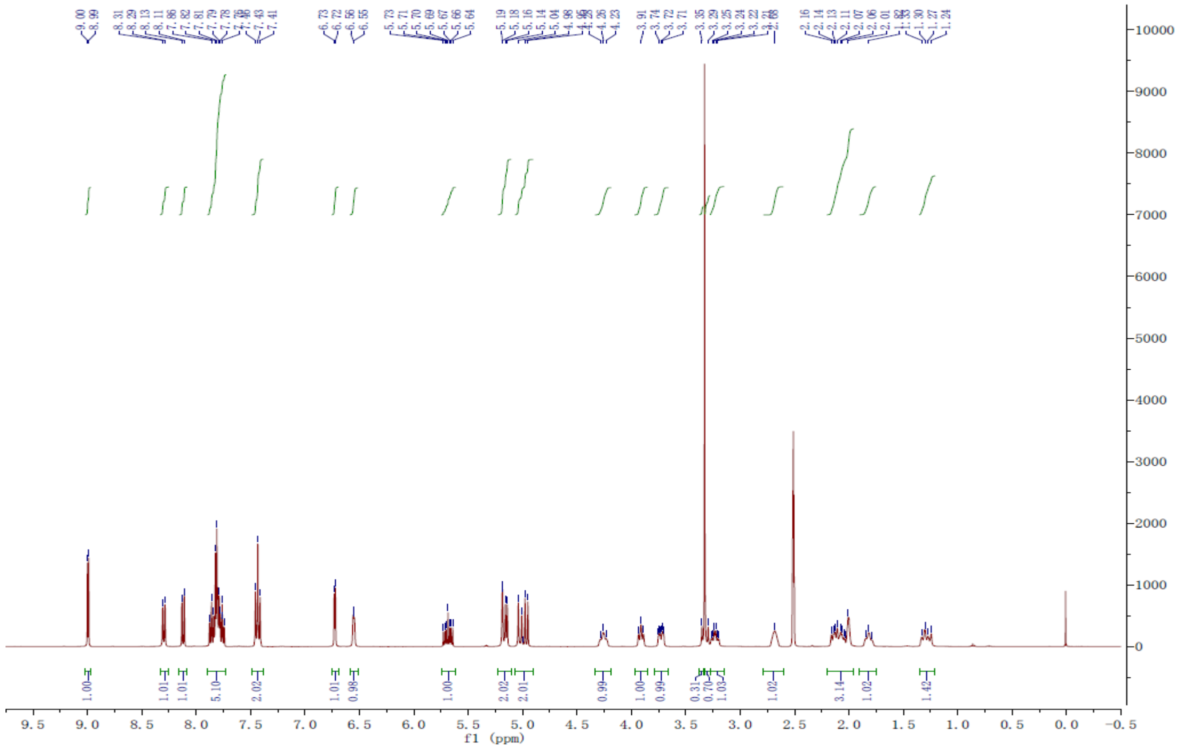

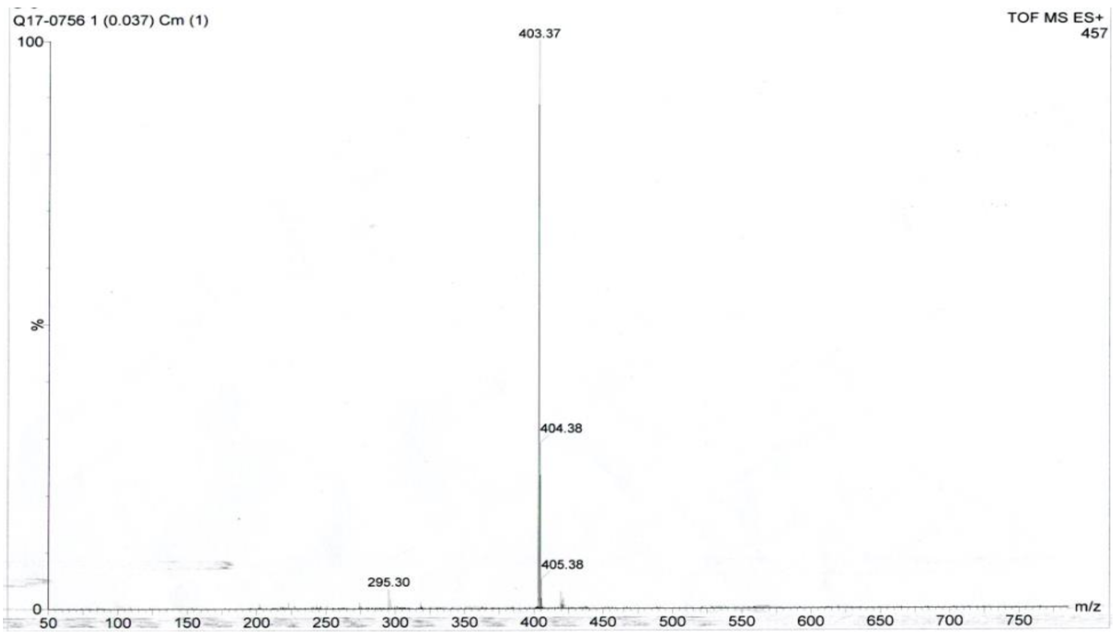

Chiral HPLC diagrams of 3 in catalysis of C6

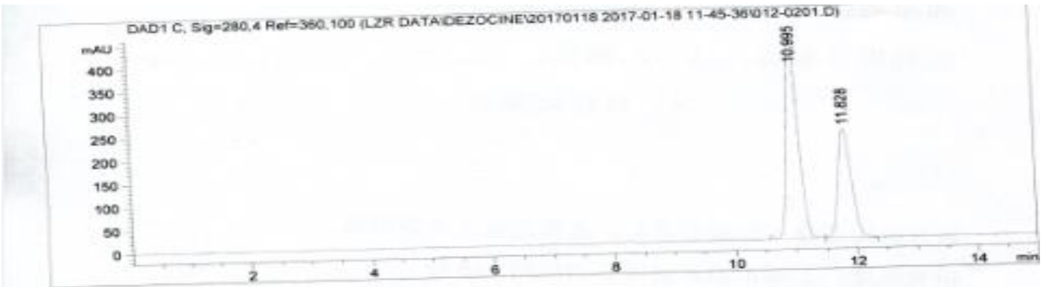

Area Percent Report

Sorted By : Signal
Multiplier : 1.0000
Dilution : 1.0000
Use Multiplier & Dilution Factor with ISTDs

Signal 1: DAD1 C, Sig=280,4 Ref=360,100

Peak #	RetTime [min]	Type	Width [min]	Area [mAU*s]	Height [mAU]	Area %
1	10.995	BB	0.2116	5929.36865	438.94278	62.7609
2	11.828	BB	0.2335	3518.18140	236.51576	37.2391

^{1}H NMR and MS spectra of catalyst C7.

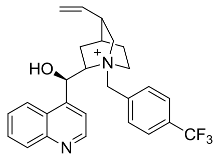

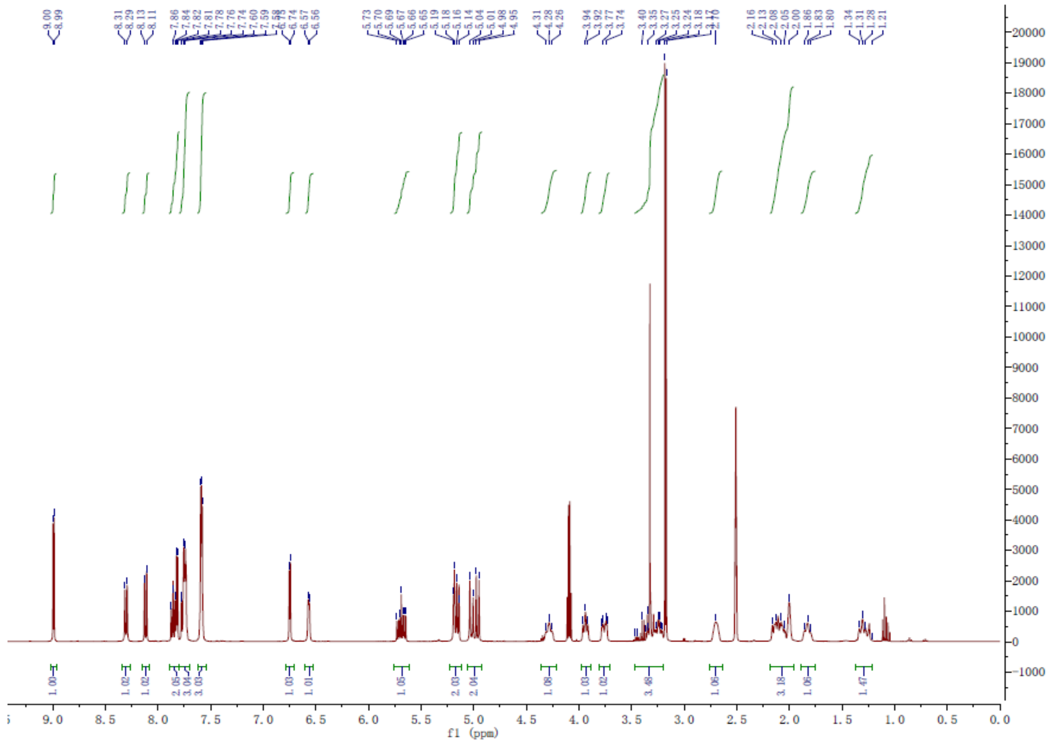

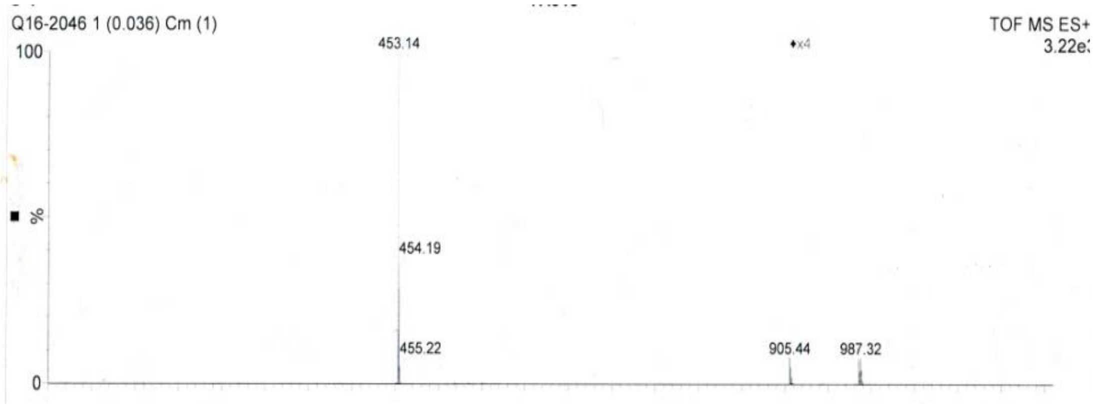

Chiral HPLC diagrams of 3 in catalysis of C7

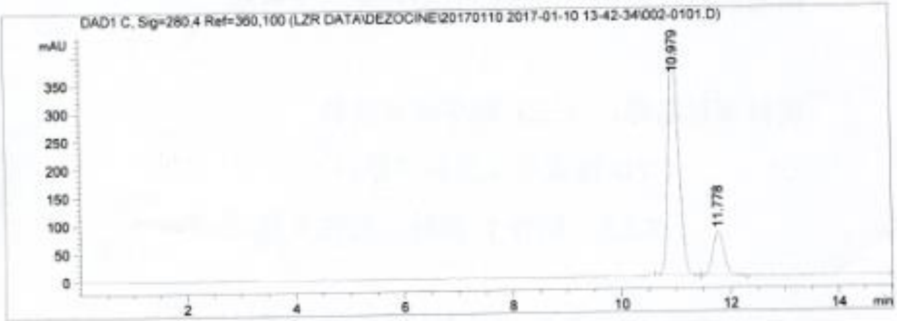


```
=====================================================================
                         Area Percent Report
=====================================================================

Sorted By             :      Signal
Multiplier            :      1.0000
Dilution              :      1.0000
Use Multiplier & Dilution Factor with ISTDs

Signal 1: DAD1 C, Sig=280,4 Ref=360,100
```

Peak #	RetTime [min]	Type	Width [min]	Area [mAU*s]	Height [mAU]	Area %
1	10.979	BB	0.2028	5389.96924	416.97266	82.9378
2	11.778	BB	0.2188	1108.84180	78.51964	17.0622

^{1}H NMR and MS spectra of catalyst C8.

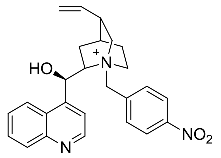

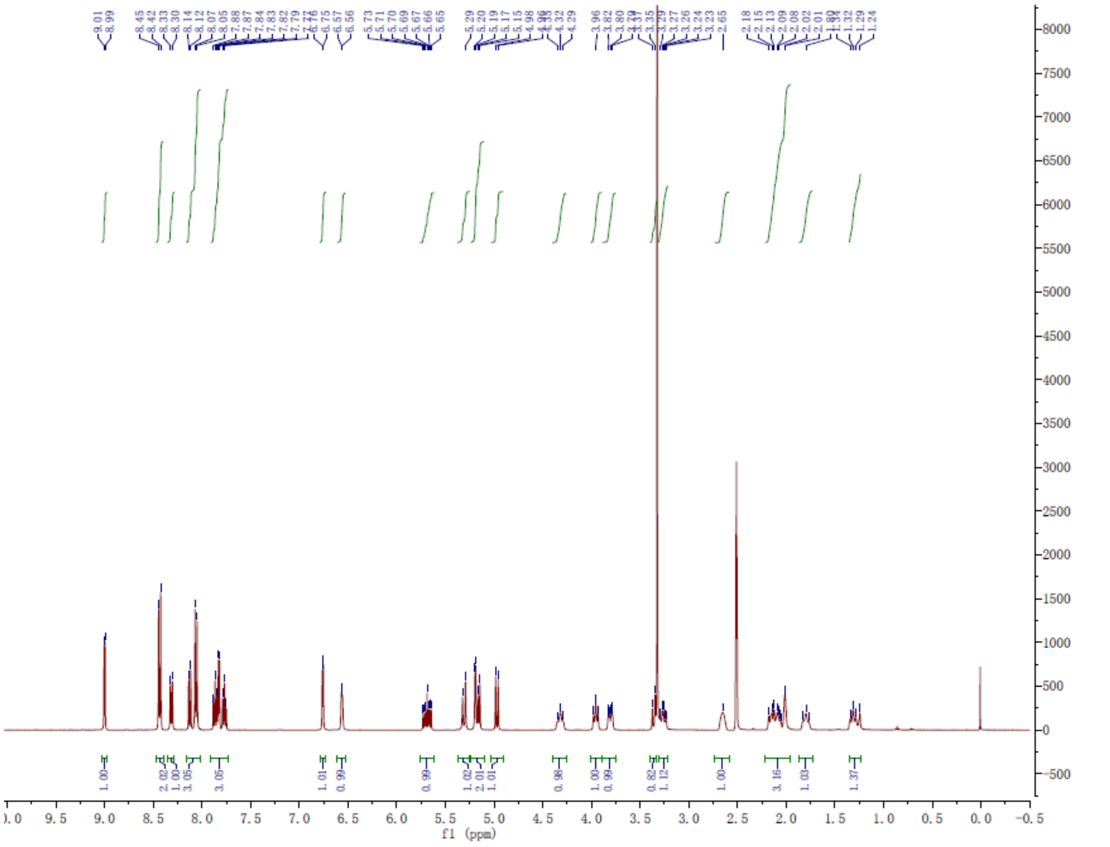

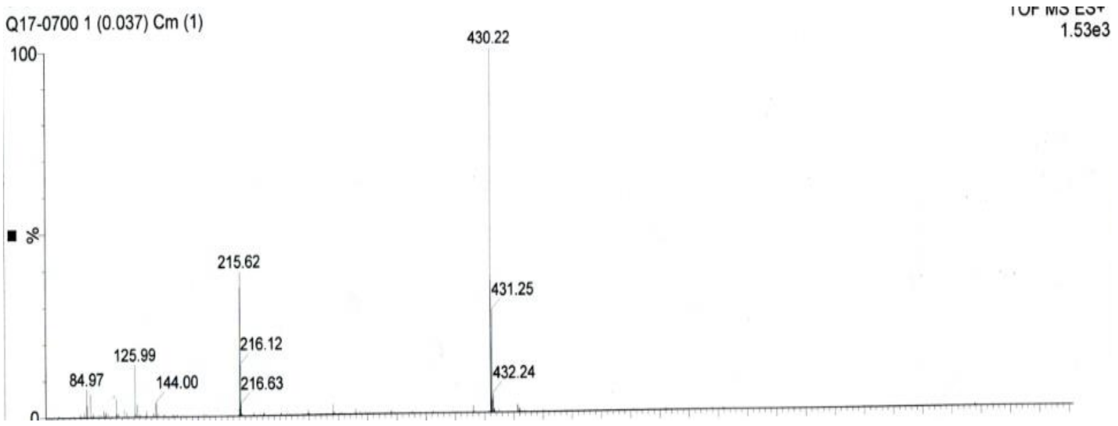

¹H NMR and MS spectra of catalyst C9.

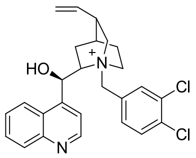

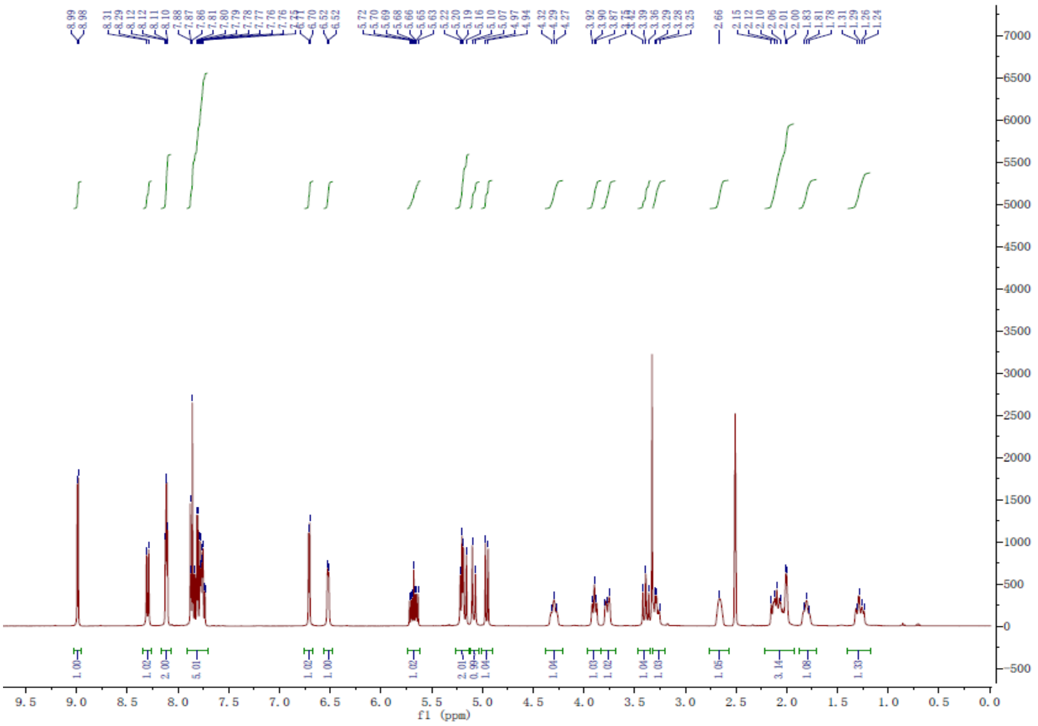

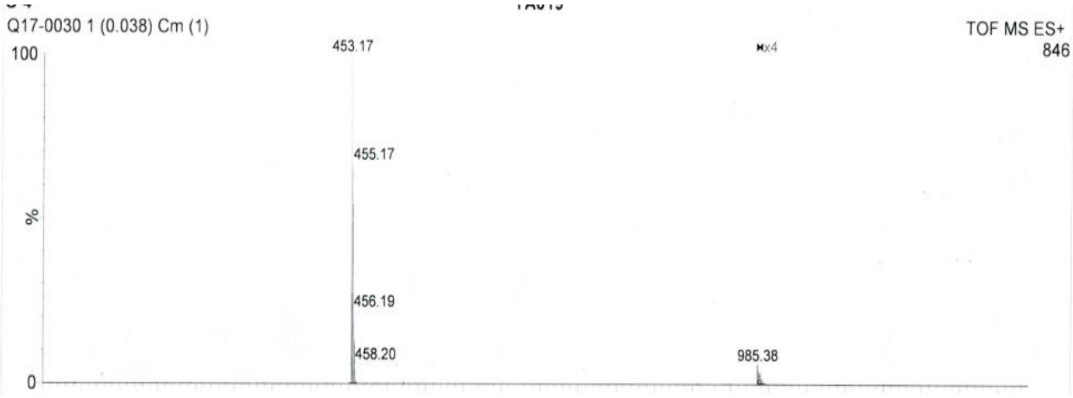

Chiral HPLC diagrams of 3 in catalysis of C9

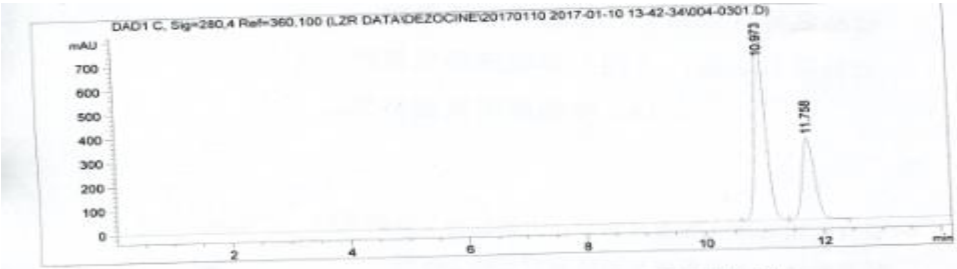

Area Percent Report

```
Sorted By                  :        Signal
Multiplier                 :        1.0000
Dilution                   :        1.0000
Use Multiplier & Dilution Factor with ISTDs

Signal 1: DAD1 C, Sig=280,4 Ref=360,100
```

Peak #	RetTime [min]	Type	Width [min]	Area [mAU*s]	Height [mAU]	Area %
1	10.973	BB	0.2043	1.02293e4	783.79688	67.5611
2	11.758	BB	0.2287	4911.52637	339.59052	32.4389

^{1}H NMR and MS spectra of catalyst C10.

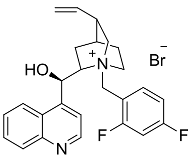

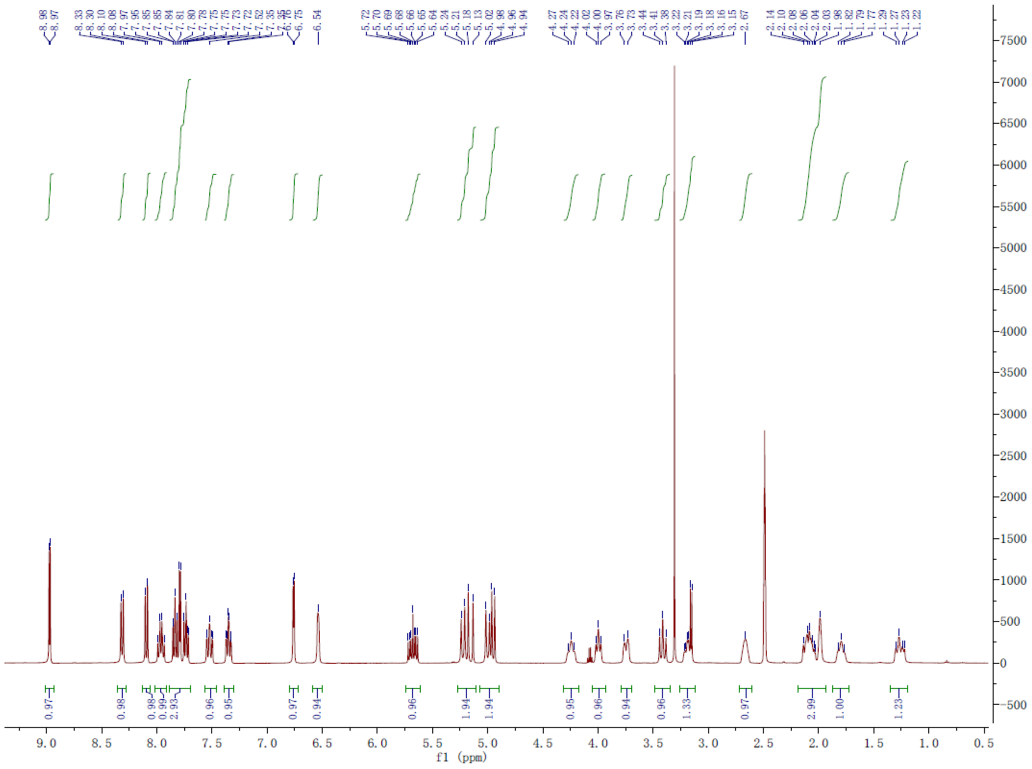

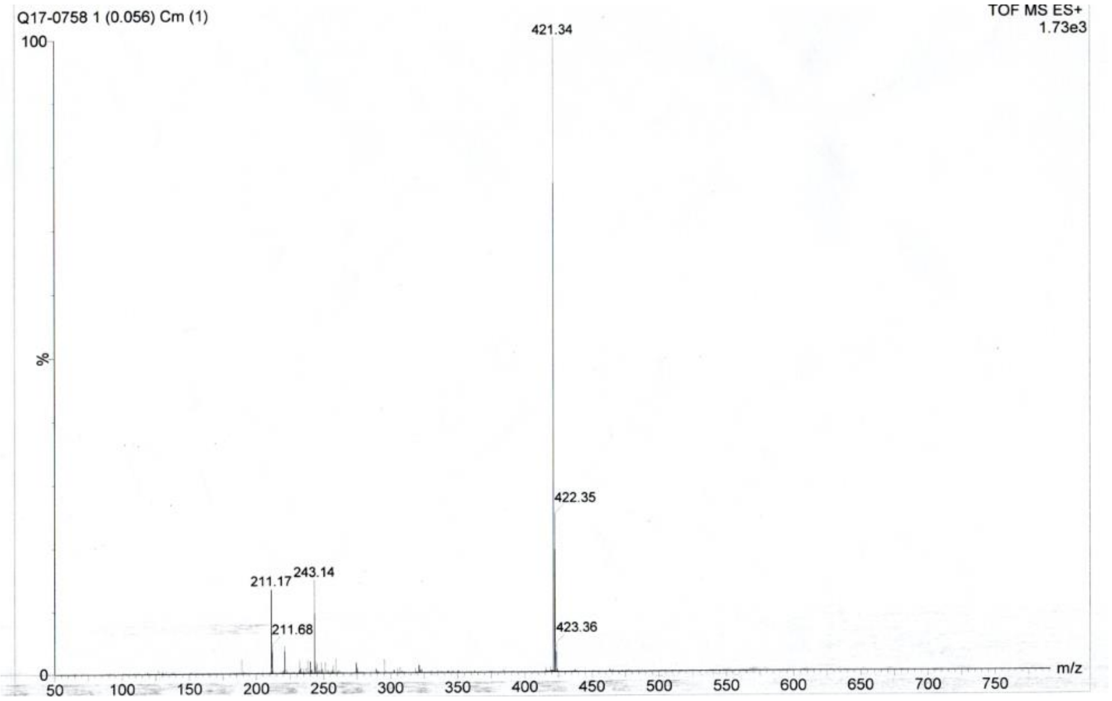

Chiral HPLC diagrams of 3 in catalysis of C10

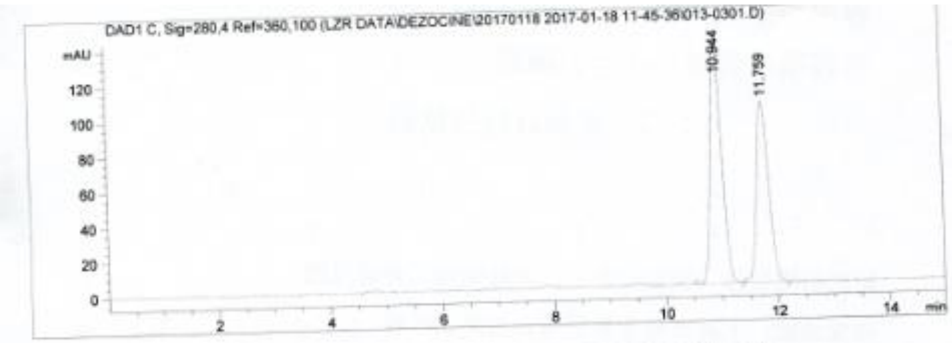

Area Percent Report

Sorted By : Signal
Multiplier : 1.0000
Dilution : 1.0000
Use Multiplier & Dilution Factor with ISTDs

Signal 1: DAD1 C, Sig=280,4 Ref=360,100

Peak #	RetTime [min]	Type	Width [min]	Area [mAU*s]	Height [mAU]	Area %
1	10.944	BB	0.2169	1898.63367	137.65794	55.1065
2	11.759	BB	0.2328	1546.75830	103.22518	44.8935

^{1}H NMR and MS spectra of catalyst C11.

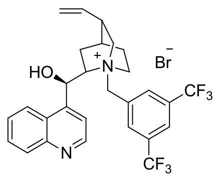

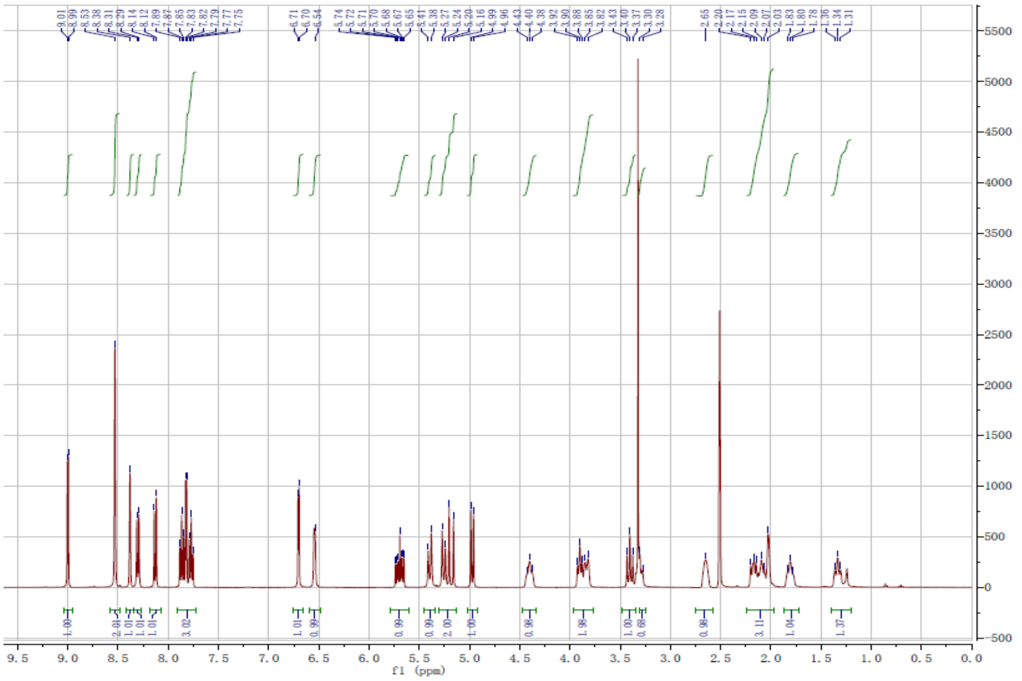

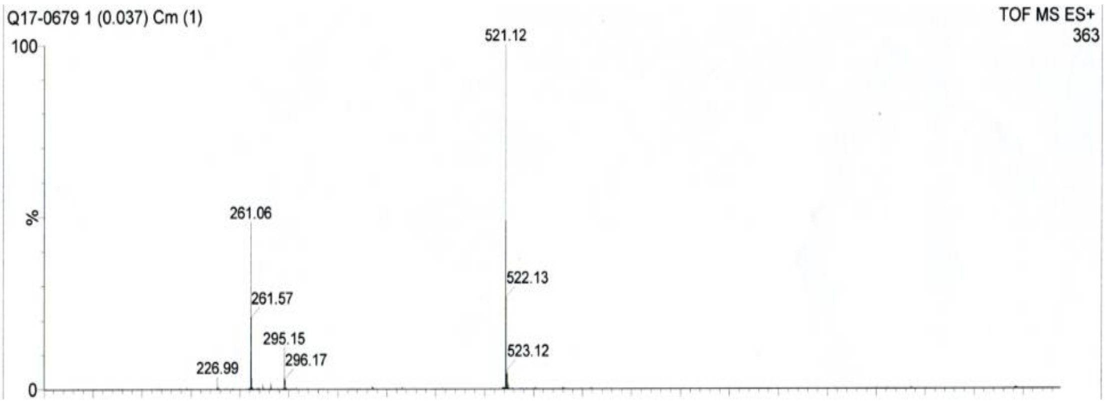

Chiral HPLC diagrams of 3 in catalysis of C11

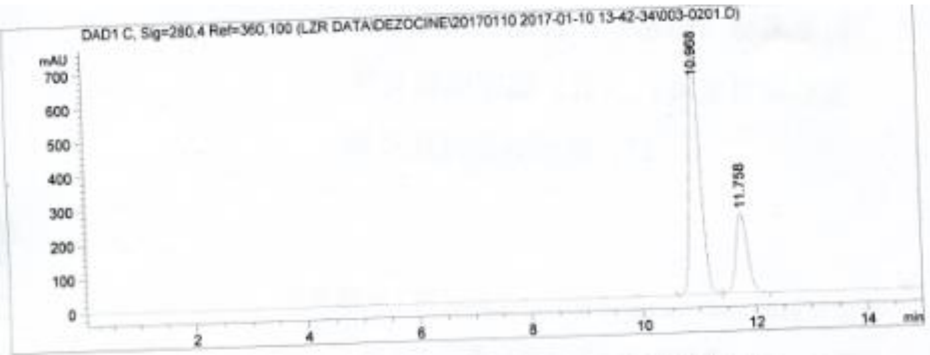

Area Percent Report

Sorted By : Signal
Multiplier : 1.0000
Dilution : 1.0000
Use Multiplier & Dilution Factor with ISTDs

Signal 1: DAD1 C, Sig=280,4 Ref=360,100

Peak #	RetTime [min]	Type	Width [min]	Area [mAU*s]	Height [mAU]	Area %
1	10.968	BB	0.2041	9595.62793	736.16815	74.0577
2	11.758	BB	0.2212	3361.32544	234.57747	25.9423

^{1}H NMR and MS spectra of catalyst C12.

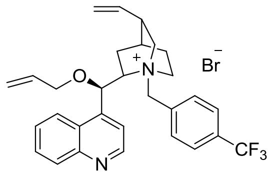

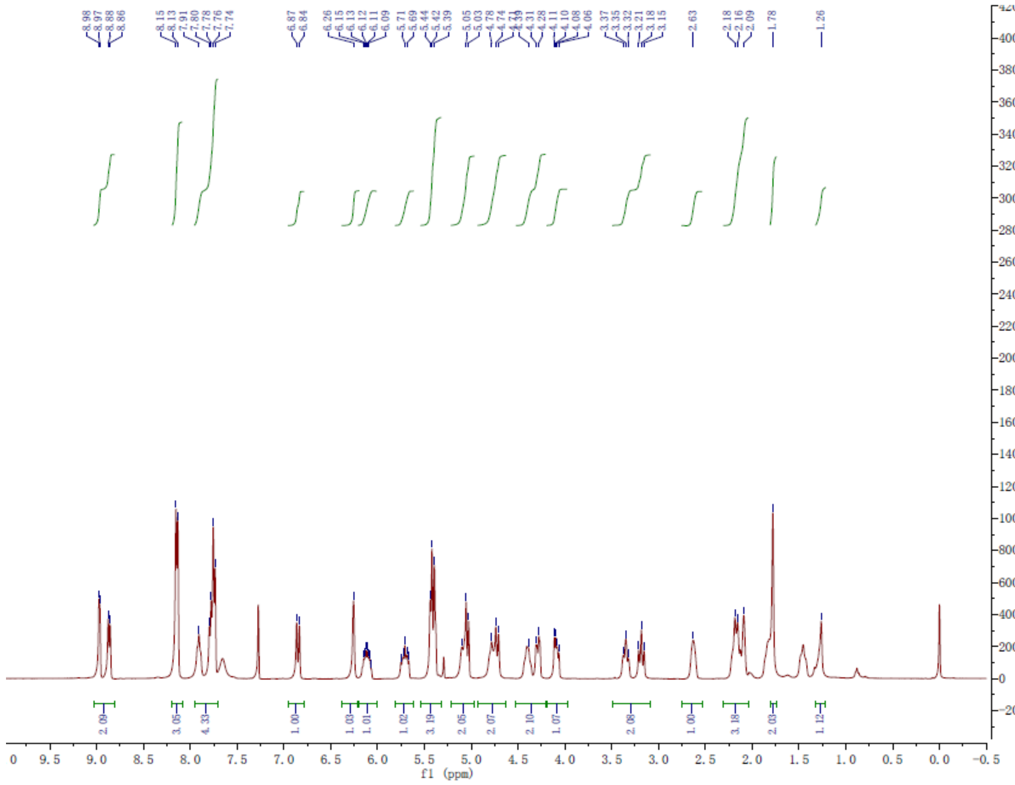

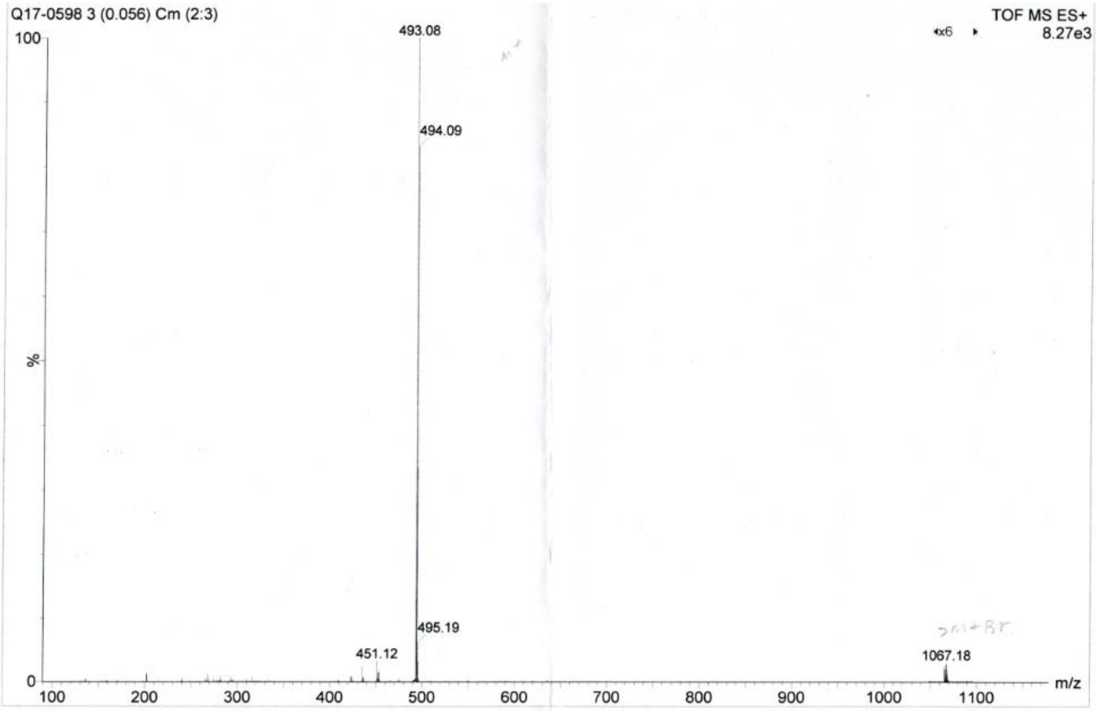

Chiral HPLC diagrams of 3 in catalysis of C12

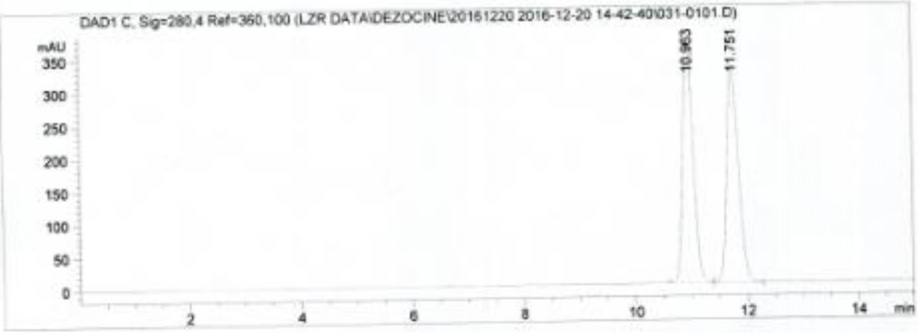

Area Percent Report

Sorted By : Signal
Multiplier : 1.0000
Dilution : 1.0000
Use Multiplier & Dilution Factor with ISTDs

Signal 1: DAD1 C, Sig=280,4 Ref=360,100

Peak #	RetTime [min]	Type	Width [min]	Area [mAU*s]	Height [mAU]	Area %
1	10.963	VV	0.2003	4744.99170	368.30511	49.9008
2	11.751	VV	0.2238	4763.86475	331.24496	50.0992

^{1}H NMR and MS spectra of catalyst C13.

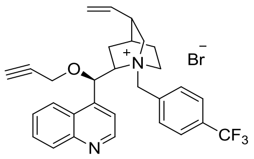

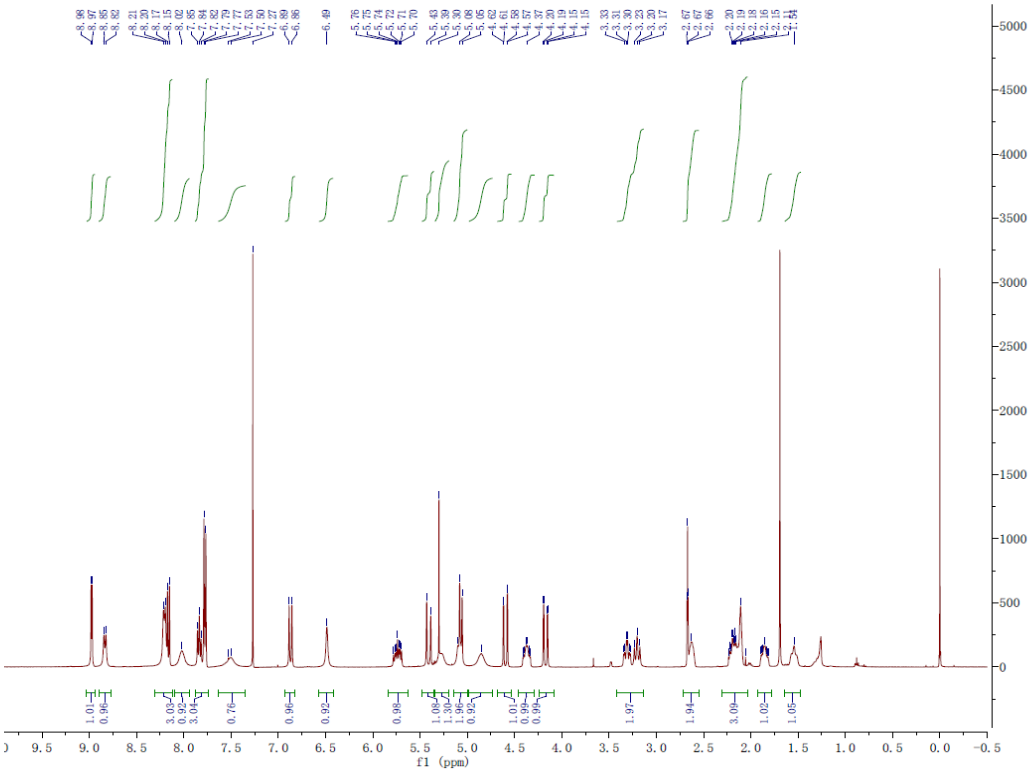

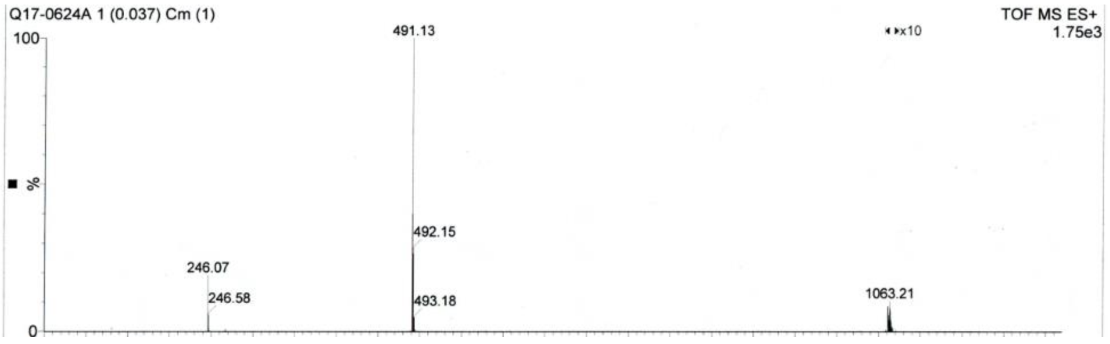

Chiral HPLC diagrams of 3 in catalysis of C13

Additional Info : Peak(s) manually integrated

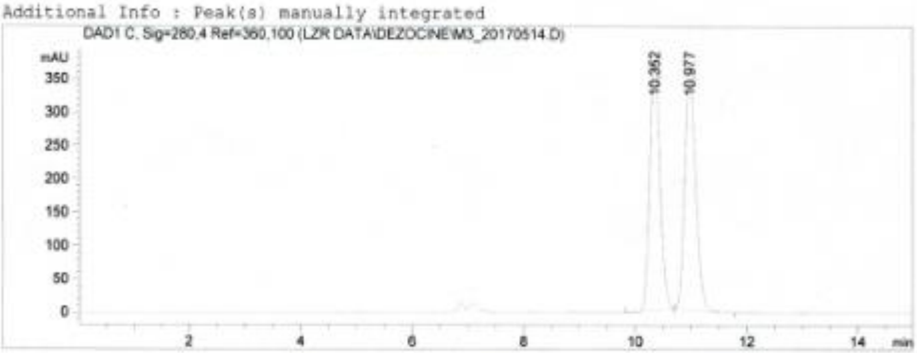

Area Percent Report

Sorted By : Signal
Multiplier : 1.0000
Dilution : 1.0000
Use Multiplier & Dilution Factor with ISTDs

Signal 1: DAD1 C, Sig=280,4 Ref=360,100

Peak #	RetTime [min]	Type	Width [min]	Area [mAU*s]	Height [mAU]	Area %
1	10.352	BB	0.1959	4697.68115	370.48657	49.7915
2	10.977	BB	0.2134	4737.02881	342.38495	50.2085

^{1}H NMR and MS spectra of catalyst C14.

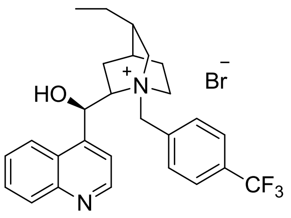

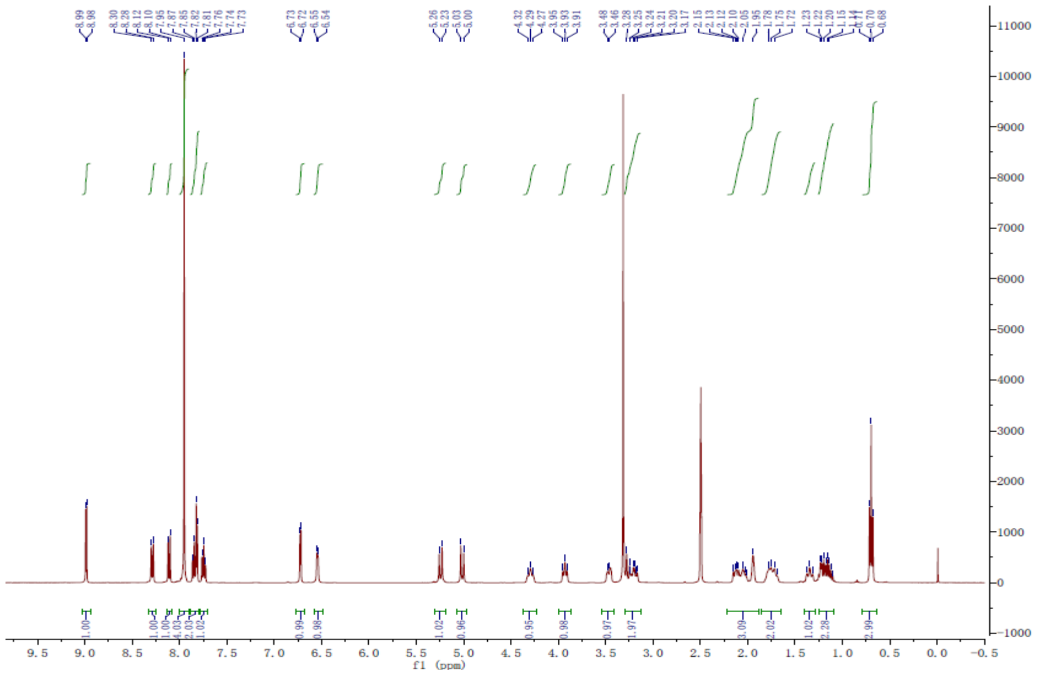

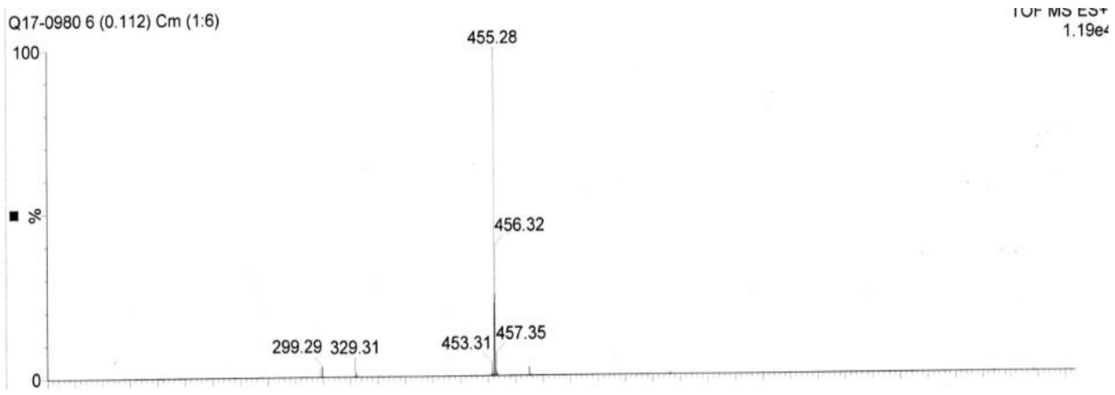

Chiral HPLC diagrams of **3** in catalysis of **C14**

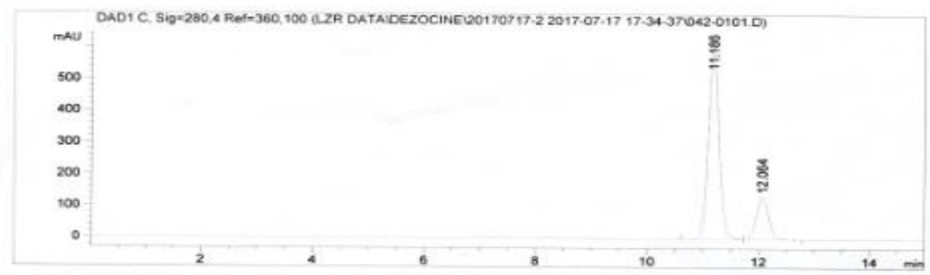


```
=====================================================================
                         Area Percent Report
=====================================================================

Sorted By             :      Signal
Multiplier            :      1.0000
Dilution              :      1.0000
Use Multiplier & Dilution Factor with ISTDs

Signal 1: DAD1 C, Sig=280,4 Ref=360,100

Peak RetTime Type  Width     Area      Height     Area
  #   [min]        [min]   [mAU*s]     [mAU]        %
----|-------|----|-------|----------|----------|--------|
  1  11.186 BB    0.1999 8020.38330  616.20020  80.0887
  2  12.064 BB    0.2302 1993.99133  135.12070  19.9113
```

^{1}H NMR and MS spectra of catalyst C15.

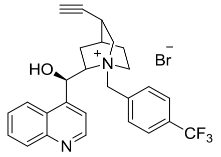

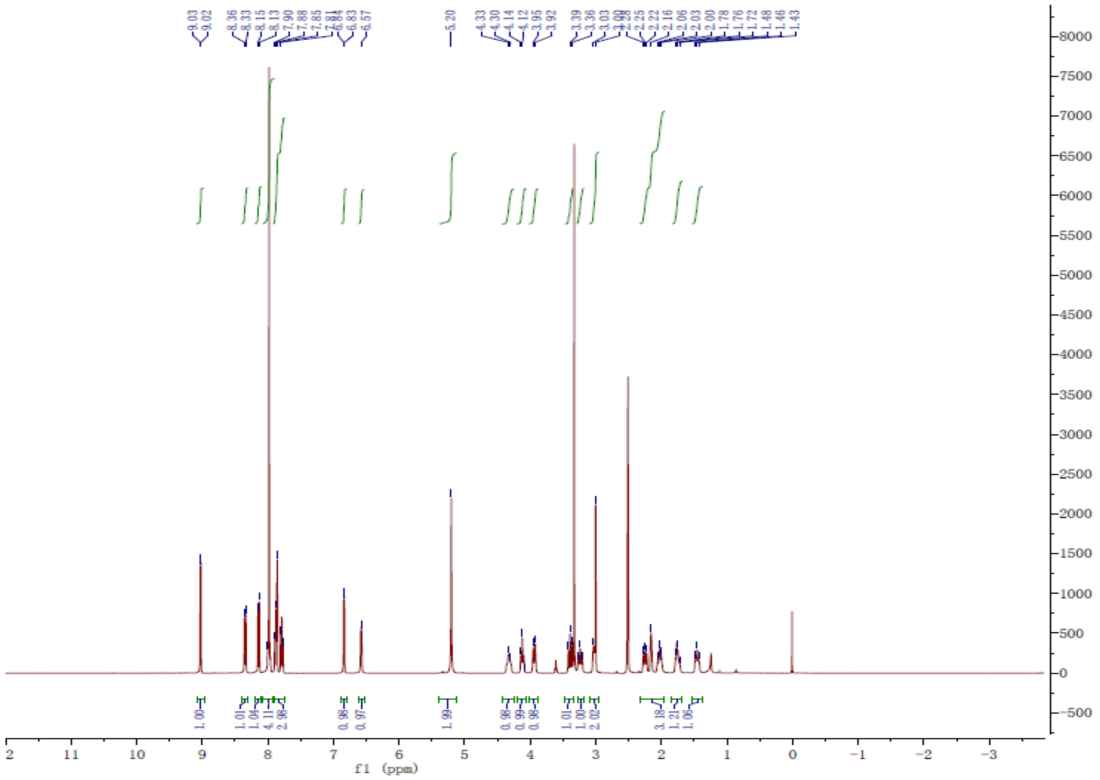

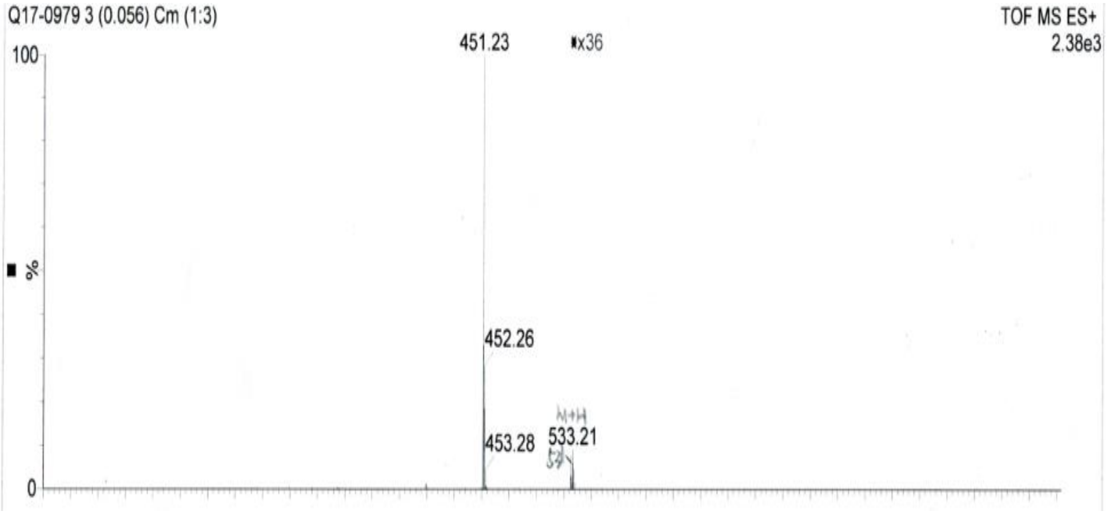

Chiral HPLC diagrams of 3 in catalysis of C15

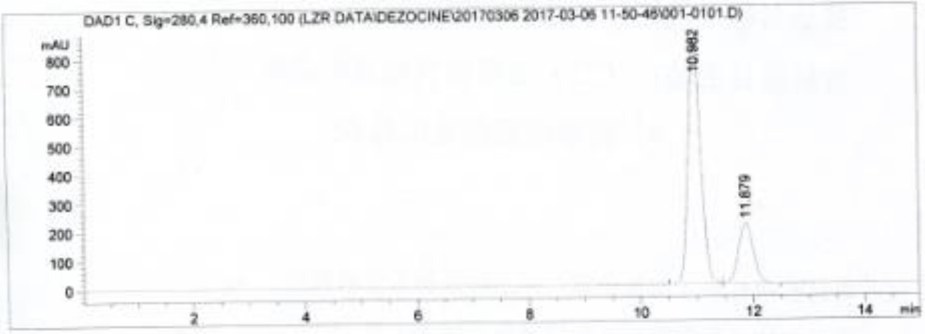

Area Percent Report

Sorted By : Signal
Multiplier : 1.0000
Dilution : 1.0000
Use Multiplier & Dilution Factor with ISTDs

Signal 1: DAD1 C, Sig=280,4 Ref=360,100

Peak #	RetTime [min]	Type	Width [min]	Area [mAU*s]	Height [mAU]	Area %
1	10.982	BB	0.2433	1.31043e4	843.42072	77.7863
2	11.879	BB	0.2781	3742.24341	209.77986	22.2137

^{1}H NMR and MS spectra of catalyst C16.

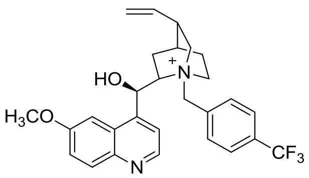

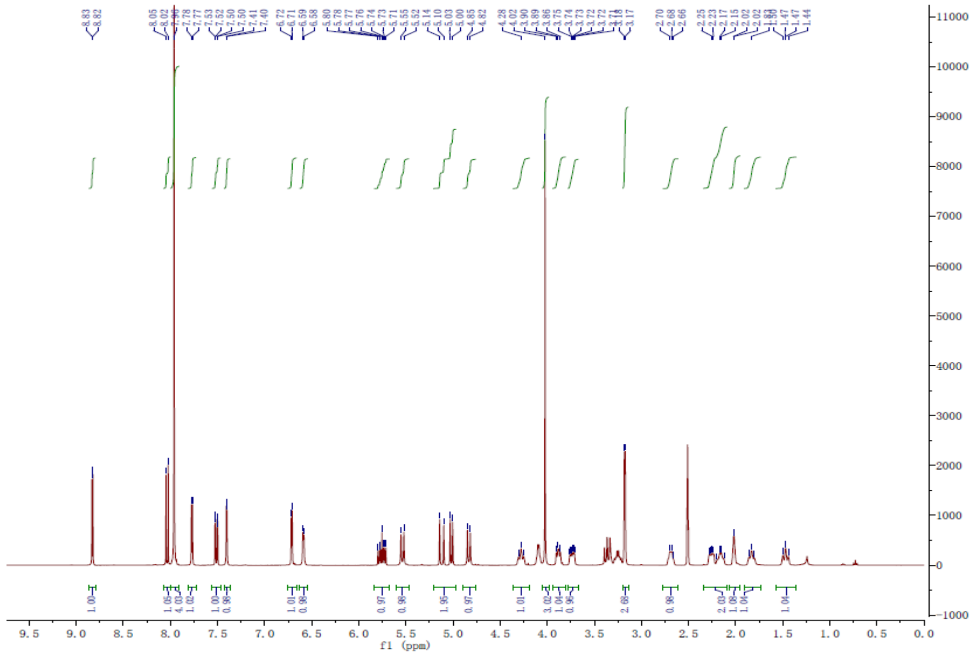

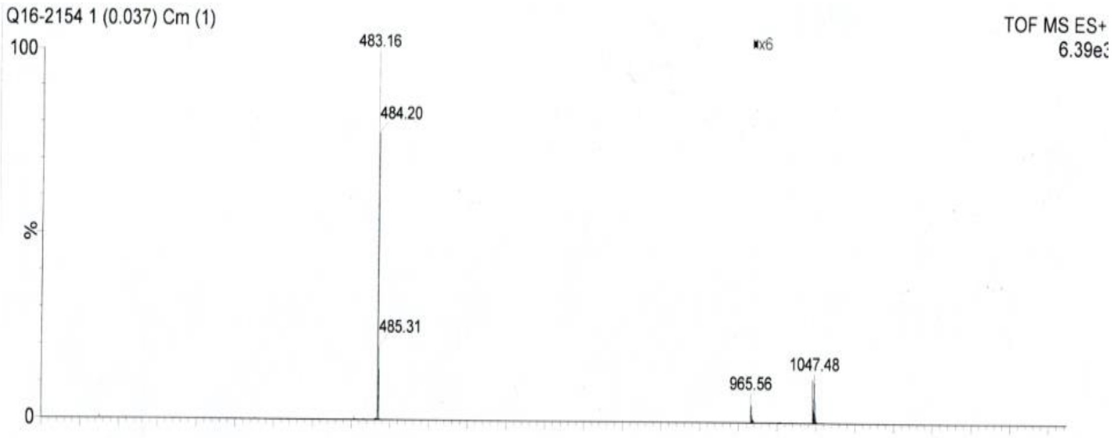

Chiral HPLC diagrams of 3 in catalysis of C16

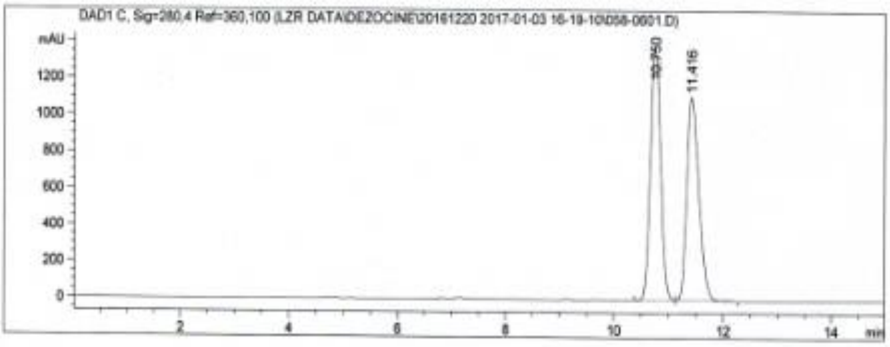

Area Percent Report

Sorted By : Signal
Multiplier : 1.0000
Dilution : 1.0000
Use Multiplier & Dilution Factor with ISTDs

Signal 1: DAD1 C, Sig=280,4 Ref=360,100

Peak #	RetTime [min]	Type	Width [min]	Area [mAU*s]	Height [mAU]	Area %
1	10.750	BB	0.2031	1.77604e4	1371.63721	51.2750
2	11.416	BB	0.2333	1.68772e4	1110.60071	48.7250

^{1}H NMR and MS spectra of catalyst C17.

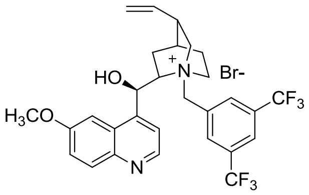

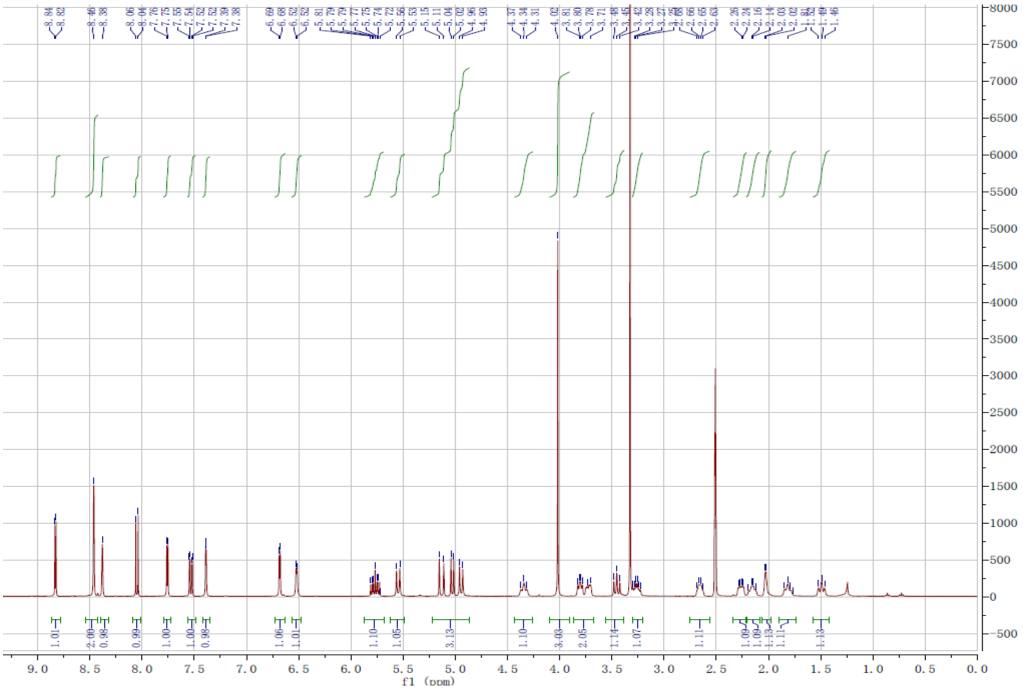

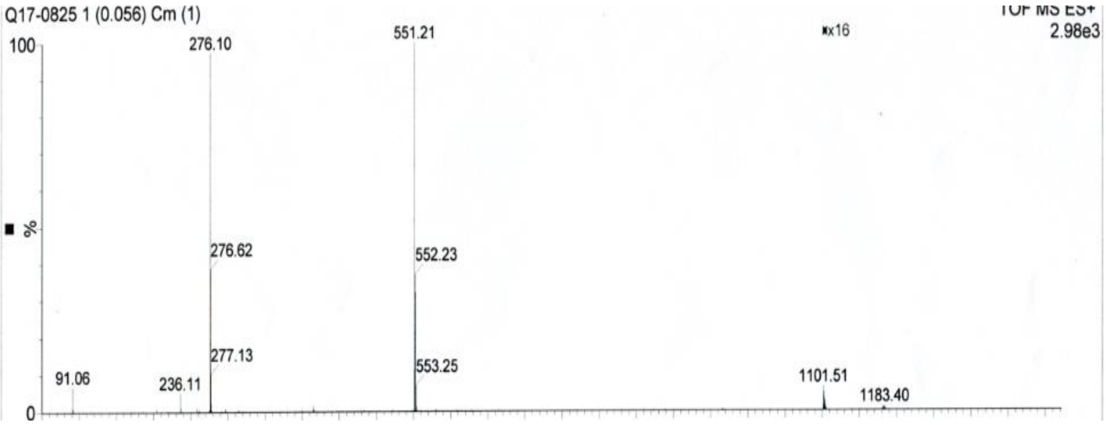

Chiral HPLC diagrams of 3 in catalysis of C17

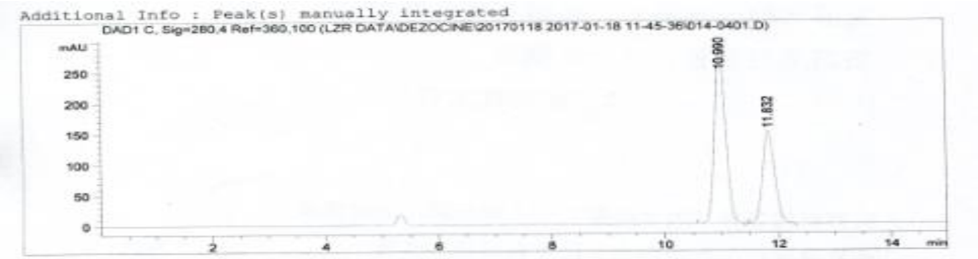

Area Percent Report

Sorted By : Signal
Multiplier : 1.0000
Dilution : 1.0000
Use Multiplier & Dilution Factor with ISTDs

Signal 1: DAD1 C, Sig=280,4 Ref=360,100

Peak #	RetTime [min]	Type	Width [min]	Area [mAU*s]	Height [mAU]	Area %
1	10.990	BB	0.2097	3849.14355	288.34256	63.6480
2	11.832	BB	0.2273	2198.40430	149.70798	36.3520

4. ^{1}H NMR, MS, ^{13}C NMR spectra of 3.

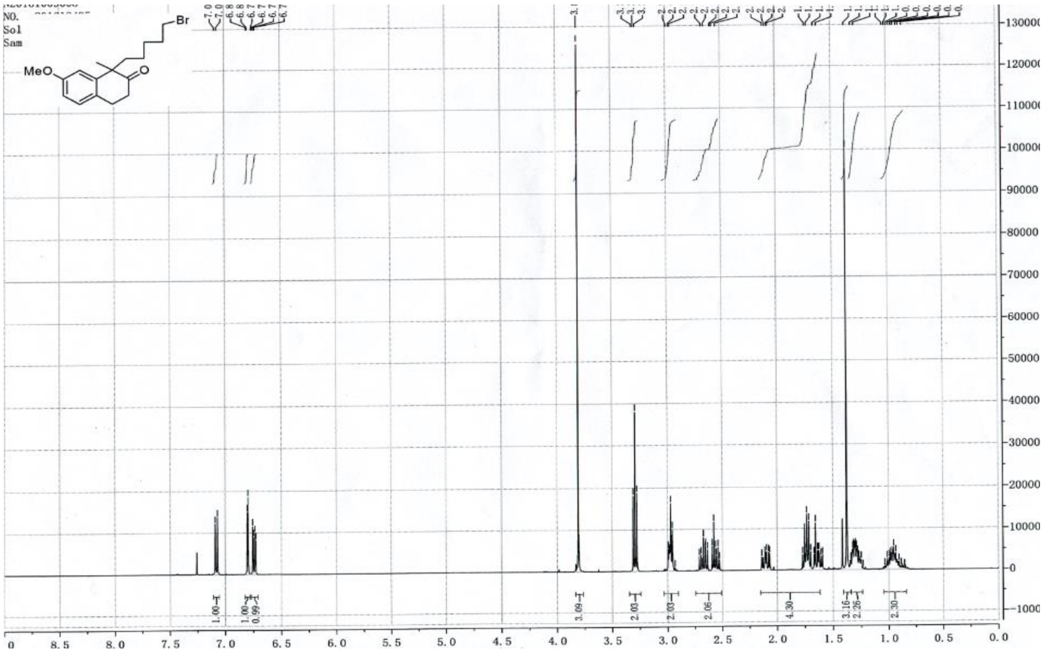

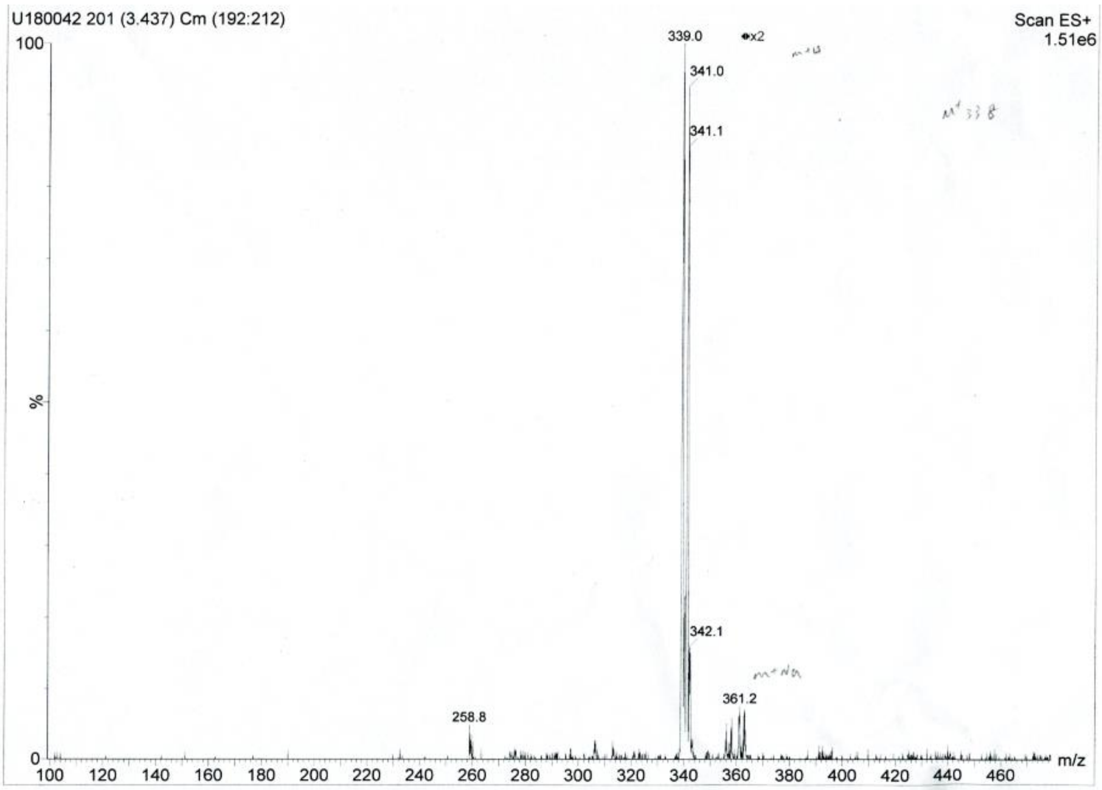
U180042 201 (3.437) Cm (192:212)
Scan ES+
1.51e6
100
%
0
339.0
341.0
341.1
342.1
361.2
258.8
m/z
100
120
140
160
180
200
220
240
260
280
300
320
340
360
380
400
420
440
460

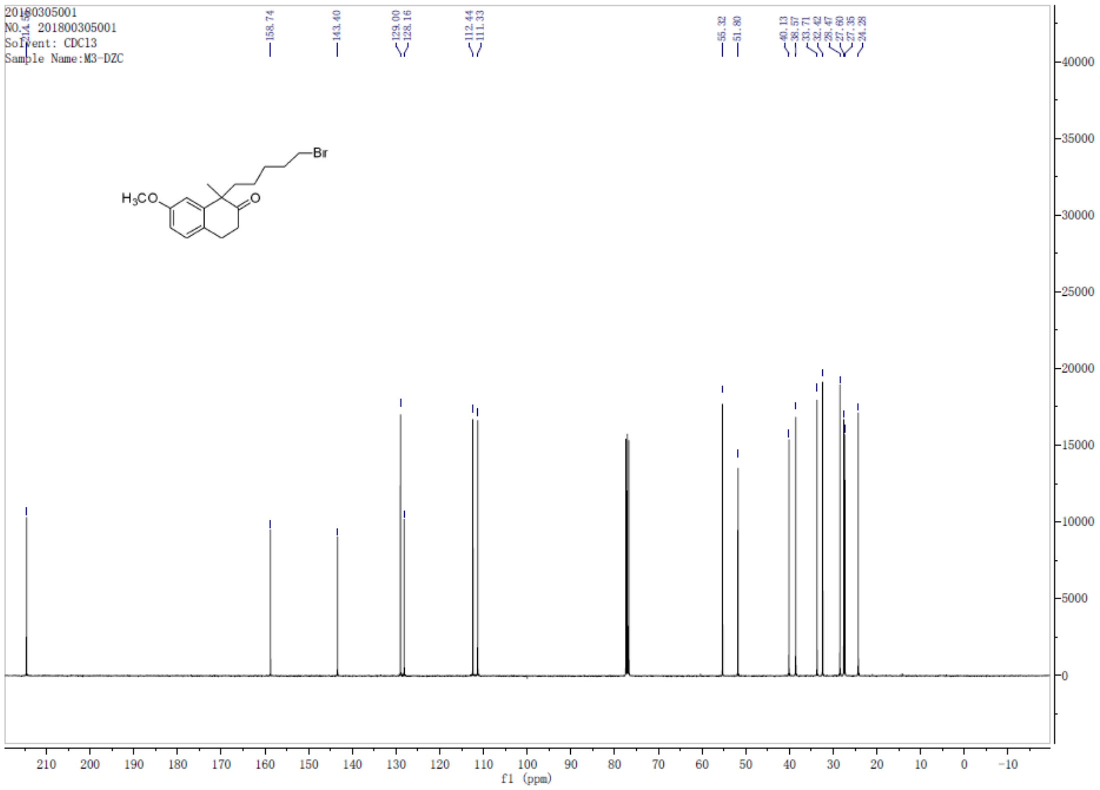
20180305001
NO. 201800305001
Solvent: CDCl3
Sample Name:M3-DZC
158.74
143.40
129.00
128.16
112.44
111.33
55.32
51.80
40.13
38.57
33.71
32.42
28.47
27.60
27.35
24.28
H3CO
Br
O
f1 (ppm)

Chiral HPLC diagrams of 3

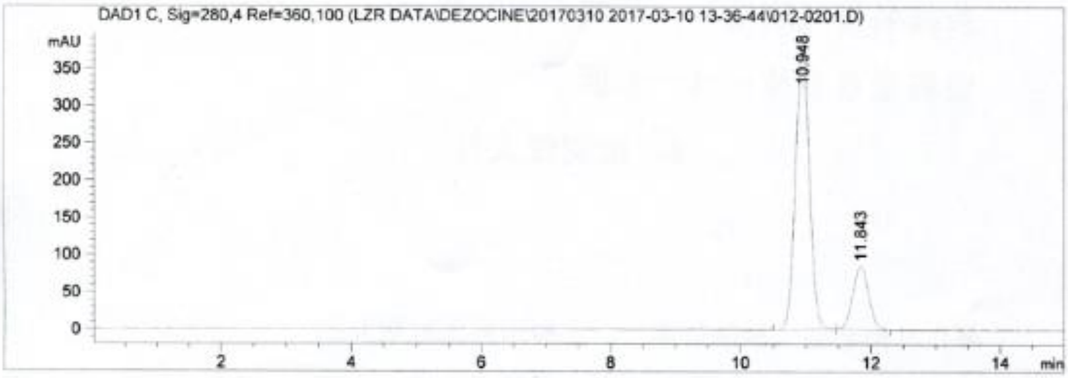


```
=====================================================================
                      Area Percent Report
=====================================================================

Sorted By             :      Signal
Multiplier            :      1.0000
Dilution              :      1.0000
Use Multiplier & Dilution Factor with ISTDs

Signal 1: DAD1 C, Sig=280,4 Ref=360,100

Peak RetTime Type  Width     Area      Height     Area
  #   [min]        [min]   [mAU*s]     [mAU]       %
----|-------|----|-------|----------|----------|--------|
   1  10.948 BB    0.2316 5619.43604  377.58997  79.6046
   2  11.843 BB    0.2648 1439.75244   85.35739  20.3954
```

5. ^{1}H NMR, MS spectra HPLC diagrams of dezocine

^{1}H NMR, MS spectra of dezocine

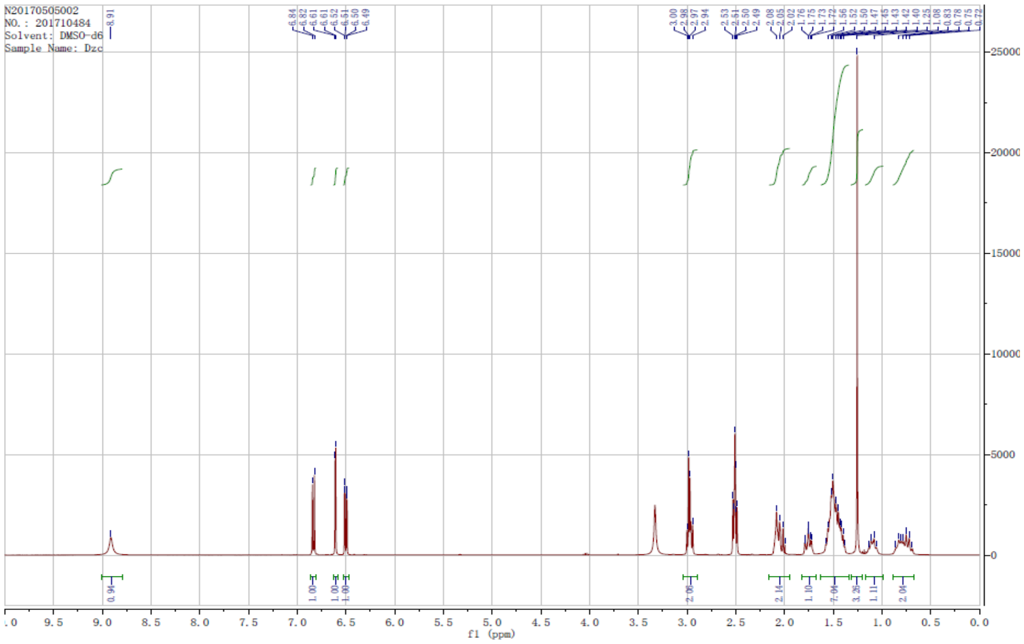

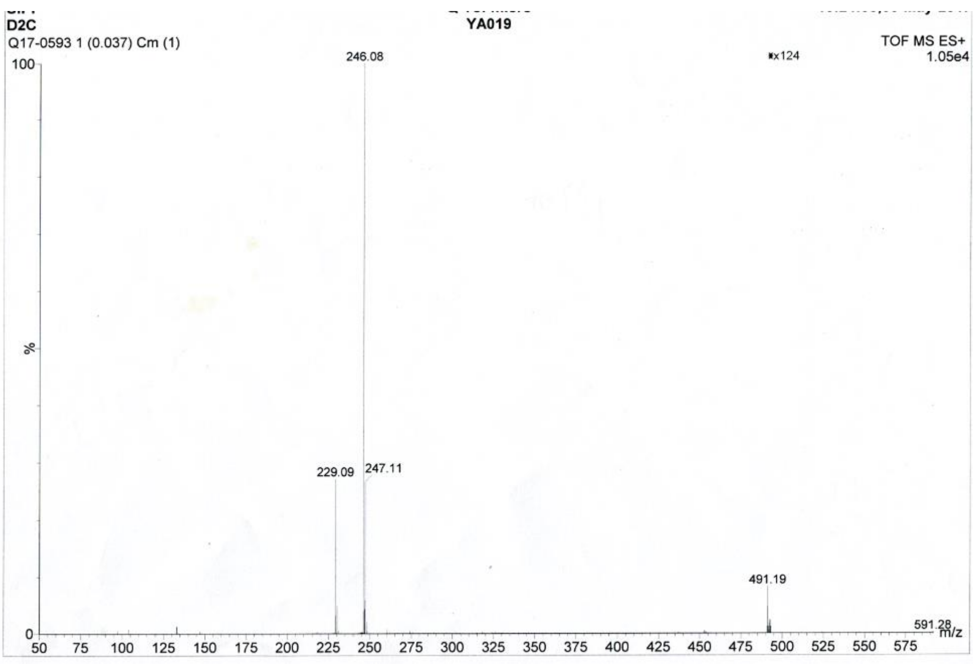

HPLC diagrams of dezocine

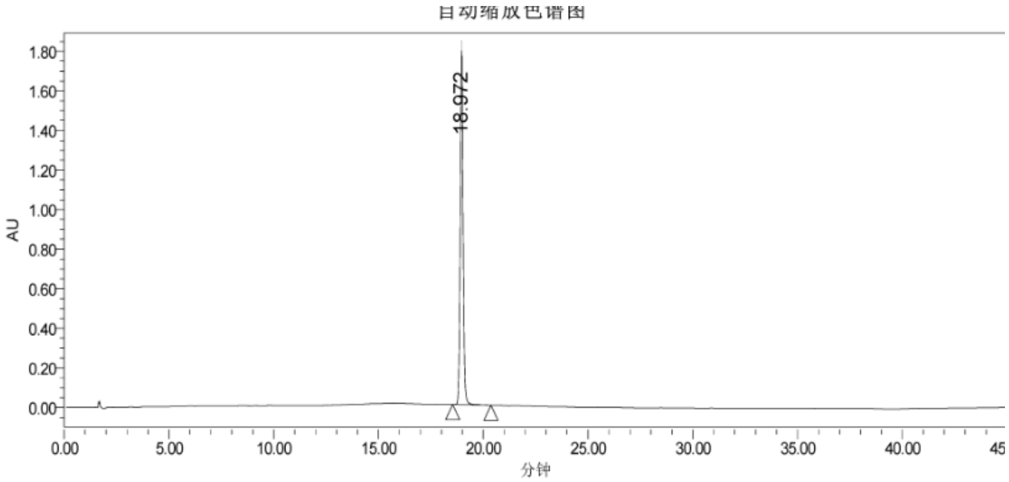

峰结果

	名称	保留时间 (分钟)	面积 (微伏*秒)	高度 (微伏)	% 面积	USP 分离度	USP 理论塔板数	对称因子
1		18.972	17005491	1787428	100.00			

Chiral HPLC diagrams of dezocine

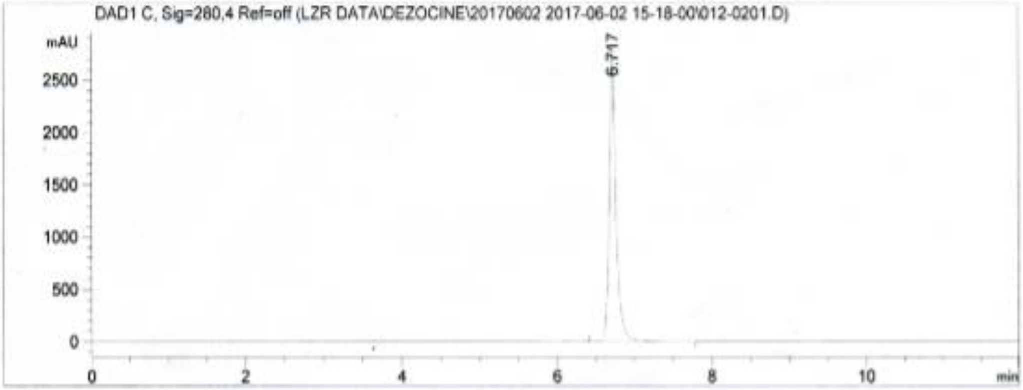

Area Percent Report

Sorted By : Signal
Multiplier : 1.0000
Dilution : 1.0000
Use Multiplier & Dilution Factor with ISTDs

Signal 1: DAD1 C, Sig=280,4 Ref=off

Peak #	RetTime [min]	Type	Width [min]	Area [mAU*s]	Height [mAU]	Area %
1	6.717	BB	0.0819	1.63661e4	2812.91260	100.0000

Totals : 1.63661e4 2812.91260
